# Engineering Nanoparticles to Modulate Extracellular Matrix and Immune Components of the Tumor Microenvironment in Cancer Immunotherapy

**DOI:** 10.34133/bmr.0289

**Published:** 2025-12-09

**Authors:** Bao-Toan Dang, Khang-Yen Pham, Ai-Han Nguyen, Jongjun Park, Taeg Kyu Kwon, Jong-Sun Kang, Jee-Heon Jeong, Simmyung Yook

**Affiliations:** ^1^ Department of Precision Medicine, School of Medicine, Sungkyunkwan University, Suwon 16419, Republic of Korea.; ^2^ Department of Biomedical Engineering, University of Connecticut, Storrs, CT 06269, USA.; ^3^ School of Pharmacy, Sungkyunkwan University, Suwon 16419, Republic of Korea.; ^4^ Department of Biopharmaceutical Convergence, Sungkyunkwan University, Suwon 16419, Republic of Korea.; ^5^ Department of Immunology, School of Medicine, Keimyung University, Daegu 42601, Republic of Korea.; ^6^ Department of Molecular Cell Biology, School of Medicine, Sungkyunkwan University, Suwon 16419, Republic of Korea.

## Abstract

Cancer immunotherapy has emerged as a transformative strategy for treating malignancies by harnessing the body’s immune system. However, its clinical efficacy is often limited by the complex and immunosuppressive nature of the tumor microenvironment (TME), which poses substantial barriers to therapeutic success. The TME comprises a variety of components, including immune cells, cancer-associated fibroblasts, abnormal vasculature, extracellular matrix, and soluble mediators that collectively support tumor progression, suppress immune surveillance, and contribute to treatment resistance and poor prognosis. Recent advances in nanotechnology have introduced engineered nanomaterials as promising tools to modulate the TME and enhance the outcomes of cancer immunotherapy. These nanomaterials can be precisely engineered to interact with specific elements of the TME, enabling localized delivery, reduced systemic toxicity, and improved therapeutic efficacy. This review provides a comprehensive overview of the role of engineered nanoparticles in targeting both cellular and noncellular components of the TME. It highlights the capacity of nanocarriers to reprogram tumor-associated immune cells, including T cells, dendritic cells, natural killer cells, and tumor-associated macrophages, as well as their ability to target cancer-associated fibroblasts, remodel tumor vasculature, degrade the extracellular matrix, and modulate immunosuppressive mediators. By exploring these multifaceted interactions, we illuminate how rationally designed nanomaterials can reshape the tumor landscape to restore immune function and enhance immunotherapeutic efficacy. Finally, the review addresses current challenges, safety considerations, and future directions necessary to translate these innovations into clinically viable therapies.

## Introduction

Nanoparticle (NP)-mediated modulation of the tumor immune landscape has rapidly evolved over recent years, driven by advances in nanotechnology and immuno-oncology [1]. Early efforts focused on improving targeted delivery of immunostimulatory agents to tumors, while more recent multifunctional NPs have enabled simultaneous delivery of combination therapeutics, immune checkpoint inhibitors, and imaging agents [2]. Current cancer therapies often overlook critical challenges such as NP heterogeneity, in vivo stability, immune-related adverse effects, and the complex interactions within the tumor microenvironment (TME) that influence therapeutic efficacy. Moreover, existing reviews tend to emphasize successful outcomes without fully addressing the translational barriers or standardized characterization methods required for clinical application. In this context, our work aims to fill these knowledge gaps by providing a comprehensive analysis of the limitations in current NP strategies and the main hurdles within the TME that inhibit the treatment efficacy of cancer immunotherapeutics, as well as by proposing innovative design principles to overcome these barriers, thereby advancing the precision modulation of the TME in cancer immunotherapy.

### Tumor microenvironment

Cancer remains a substantial and growing global public health challenge, accounting for roughly 1 in every 6 deaths worldwide [3]. In response, a diverse array of therapeutic strategies has been introduced, encompassing surgery, radiotherapy, chemotherapy, targeted therapy, and immunotherapy [4]. Among these, immunotherapy has gained attention as a particularly innovative and promising modality, offering the potential for long-lasting and highly specific anticancer effects. In contrast to traditional treatments, immunotherapy activates the body’s immune system to detect and destroy malignant cells, relying on the intricate interplay between tumor biology and host immune surveillance mechanisms [5].

The various ecosystems that constitute a permissive TME underscore the complexity of tumors. A detailed examination of the TME reveals a network of cellular and noncellular components that generate signals regulating tumor cell survival, proliferation, angiogenesis, immune evasion, and metastasis [6,7]. The TME landscape can be broadly categorized into 3 ecosystems: (a) the cellular compartment, (b) the soluble components, and (c) the extracellular matrix (ECM). The tumor niche is a highly dynamic 3-dimensional (3D) structure in which stromal cells play a critical role in regulating distinct phases of tumor development, alongside intricate interactions among these cells [8,9]. Importantly, while TMEs across various tumor types or subtypes exhibit common traits, each also displays distinct characteristics, creating a supportive “ecosystem” for tumor growth and development [10].

Recent research categorizes the TME into “hot” and “cold” types based on the level of immune cell infiltration [11]. Hot TME contain high numbers of proinflammatory immune cells, such as M1 macrophages and CD8^+^ T cells, and are generally associated with better prognosis, longer survival, and greater responsiveness to immunotherapy [12]. In contrast, cold TMEs are dominated by immunosuppressive cells, including M2 macrophages, regulatory T cells (Tregs), and myeloid-derived suppressor cells (MDSCs), and are usually linked to poorer clinical outcomes [13]. As a result, strategies aimed at converting cold TME into hot ones remain a key challenge and a central objective in cancer immunotherapy.

In general, cancer and TME are closely interconnected. Cancer cells constantly interact with and remodel the TME, which in turn substantially affects tumor cell behavior. Because of this central role, the TME strongly influences drug delivery, therapeutic resistance, and overall clinical outcomes [14]. Therefore, targeting and modulating the TME has become a crucial strategy for overcoming treatment resistance and potentially altering the course of cancer progression.

### Hurdles of TME in cancer therapy

Cancer immunotherapy has emerged as a compelling strategy for treating multiple malignancies, offering the potential for durable clinical responses and long-term remission. Yet, its therapeutic efficacy continues to be limited by several key challenges. These include the intrinsically low immunogenicity of tumor cells, systemic toxicities, and, most substantially, the profoundly immunosuppressive characteristics of the TME [15,16].

The TME is a pivotal driver of cancer progression and therapeutic resistance. It comprises not only tumor cells but also a diverse and dynamic network that includes immune cells (e.g., T cells, macrophages, and Tregs), fibroblasts, endothelial cells, ECM, chemokines, and hypoxic zones. This complex and heterogeneous environment profoundly influences tumor proliferation, immune evasion, and metastatic behavior [17,18].

**Fig. 1.**
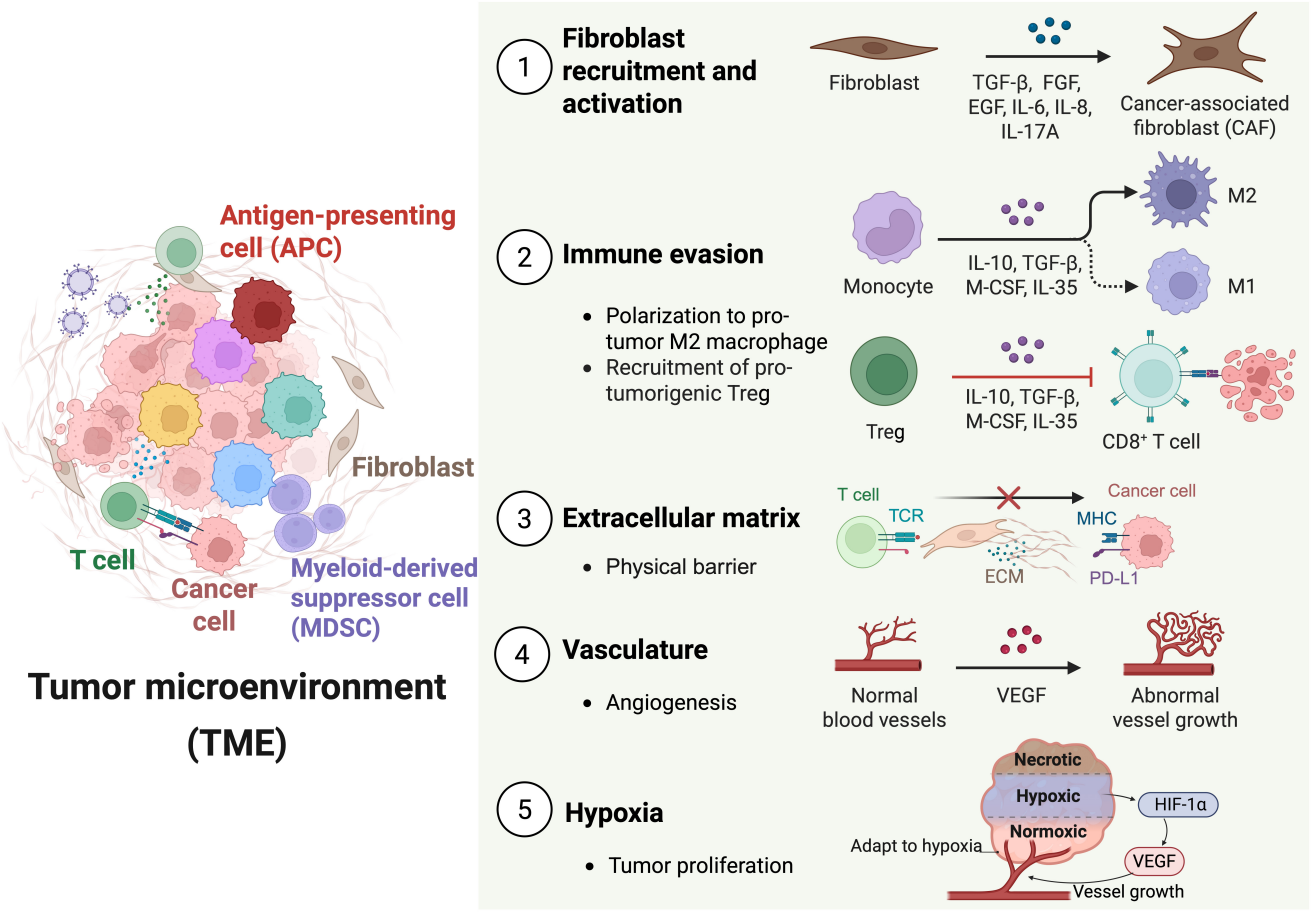
Immunosuppressive components and barriers within the TME that hinder cancer immunotherapy. Key immunosuppressive mechanisms include the following: (1) Fibroblast recruitment and activation—fibroblasts are converted into CAFs through cytokines such as TGF-β, FGF, IL-6, IL-8, and IL-17A, promoting tumor growth. (2) Immune evasion—monocytes polarize into protumorigenic M2 macrophages and Tregs are recruited, both secreting IL-10, TGF-β, M-CSF, and IL-35 to suppress CD8^+^ T cell activity. (3) ECM—the dense ECM forms a physical barrier to immune infiltration, while tumor cells exploit immune checkpoint interactions to evade T cell recognition. (4) Vasculature—abnormal angiogenesis driven by VEGF results in irregular, leaky vessels that impair immune cell trafficking and drug delivery. (5) Hypoxia—hypoxic conditions within the TME, mediated by HIF-1α, stimulate VEGF expression, enhance angiogenesis, and support tumor proliferation and survival. Created with BioRender.

Multiple components within the TME substantially contribute to immunotherapy resistance through diverse mechanisms (Table S1) [6]. First, cancer-associated fibroblasts (CAFs) promote therapeutic resistance by secreting a variety of chemokines, metabolites, and growth factors, including interleukin-17A (IL-17A), IL-6, E74-like factor (ELF), fibroblast growth factor 5 (FGF5), hepatocyte growth factor (HGF), stanniocalcin 1 (STC1), insulin-like growth factor binding protein 3 (IGFBP3), and transforming growth factor-β2 (TGF-β2) [19]. These soluble factors create an environment that supports tumor survival and impairs immune cell function. Second, immune cells within the TME also play a key role by sustaining an immunosuppressive milieu that undermines therapeutic effectiveness. This includes the involvement of regulatory and effector T cells, cytotoxic T lymphocytes (CTLs), tumor-associated macrophages (TAMs), and MDSCs [20]. Third, the ECM, composed of structural proteins such as laminin, elastin, and collagen, offers both physical support and biochemical cues to tumor and stromal cells. The ECM facilitates immune evasion by forming a physical barrier that hinders immune cell infiltration and impedes the penetration of therapeutic agents [21]. Fourth, the abnormal vasculature characteristic of tumors further reduces treatment efficacy by disrupting the supply of nutrients and oxygen. This defective vascularization limits drug delivery and fosters acidic, hypoxic conditions that contribute to resistance [22]. Finally, hypoxia, driven by vascular abnormalities and heightened oxygen demand, activates hypoxia-inducible factor 1 (HIF-1), which enhances tumor proliferation, adaptation to low-oxygen conditions, and resistance to therapy. HIF-1 also up-regulates resistance-associated proteins such as P-glycoprotein and dihydrofolate reductase, thereby complicating the success of treatments like topoisomerase II inhibitors [23,24] (Fig. 1).

Moreover, tumors actively remodel the TME to evade immune surveillance by promoting immunosuppressive conditions. Recent studies have emphasized that various tumors can modulate immune responses through mechanisms such as immunoediting [25]. This process entails the suppression of effective immune responses and the enhancement of tumor-promoting elements like TAMs, Tregs, and MDSCs. Simultaneously, CTL activity is frequently diminished through apoptosis or functional exhaustion, and overall lymphocyte levels may decline [12,26]. Additionally, several immunosuppressive factors within the TME, including prostaglandin E2, vascular endothelial growth factor (VEGF), IL-10, and TGF-β, have been shown to directly inhibit antitumor immune responses. Tumor cells may also disrupt immune equilibrium by down-regulating T helper 1 (Th1)-type responses, which are vital for CTL activation, while promoting Th2 responses that are less effective in tumor elimination [7].

Given these complexities, there is growing recognition that targeting immune checkpoint pathways alone is inadequate. Successful cancer immunotherapy must also counteract the immunosuppressive forces exerted by the TME. Targeting specific components within the TME offers a promising strategy to resensitize resistant tumors and improve the overall effectiveness of immunotherapeutic interventions [27]. Thus, a deeper understanding of the dynamic interplay between tumor cells and their microenvironment is critical for the development of next-generation cancer immunotherapies.

### NPs modulating TME in cancer immunotherapy

Nanomedicine offers a groundbreaking platform for cancer immunotherapy by facilitating the precise, localized delivery of therapeutic agents directly into the TME. This targeted strategy reduces systemic toxicity while enhancing antitumor efficacy [28,29]. Tumors often exploit physiological pathways, such as those involved in wound healing and immune modulation, to support growth and suppress immune activity. For instance, eliminating Tregs may alleviate immunosuppression within the TME, but systemic depletion risks broader immune disruption, including autoimmunity and increased vulnerability to infections [7,30]. To address these limitations, NPs have been designed to selectively modulate the TME while maintaining systemic immune homeostasis. In recent years, NP-based delivery strategies have increasingly targeted the TME to inhibit tumor progression and survival. One such approach involves the “repolarization” of immunosuppressive M2 TAMs toward the M1 phenotype, which possesses antitumor activity and can trigger immune responses against cancer cells [31]. However, outcomes have varied due to differences in patient characteristics, tumor heterogeneity, and the complex nature of the TME [32]. These systems are typically functionalized with ligands such as antibodies, peptides, or small molecules that recognize specific markers on tumor or immune cells, enabling localized and cell-specific delivery of immunomodulatory agents [33,34].

Compared to conventional antibody-based therapies, NPs offer distinct advantages. Their structural versatility allows for the encapsulation of a wide range of therapeutic agents, including hydrophobic drugs, nucleic acids, proteins, and combination treatments [35]. In addition, NPs often exhibit higher drug-loading capacities than antibody–drug conjugates. Their surfaces can also be engineered to enhance stability, evade immune clearance, and enable controlled drug release. Antibody-conjugated NPs merge the high specificity of biological targeting with the multifunctionality of nanocarriers, yielding synergistic therapeutic benefits [36].

A key advantage of NP-mediated delivery is the potential to widen the therapeutic window by minimizing off-target toxicity. A well-known example is liposomal amphotericin B, which significantly reduces nephrotoxicity compared to its traditional formulation [37]. In cancer immunotherapy, comparable strategies aim to confine immune activation to the TME, thereby limiting systemic immune-related adverse effects. Another major benefit of nanomaterials is their enhanced ability to penetrate solid tumors, especially important in desmoplastic or poorly vascularized tissues where larger biologics often fail to achieve therapeutic levels. Advances in NP engineering, such as stimuli-responsive release and active transport mechanisms, have further improved intratumoral distribution beyond the constraints of the enhanced permeability and retention (EPR) effect [38].

As a result, NPs have shown considerable promise in reshaping the immunosuppressive TME. They can enhance immune cell infiltration, alleviate hypoxia, modulate cytokine profiles, and improve antigen presentation. Collectively, these effects increase tumor sensitivity to immune checkpoint inhibitors (ICIs) and other immunotherapies [39]. A growing body of research supports the role of nanotechnology in reprogramming the TME to promote antitumor immunity and improve therapeutic efficacy. In light of these developments, several NP-based immunotherapeutic platforms have advanced to clinical evaluation [40]. These systems exemplify the translational potential of nanotechnology bridging laboratory research and clinical application—by improving delivery precision, boosting immune activation, and reducing systemic toxicity [41].

The following section explores recent advancements in NP platforms specifically designed to modulate components of the TME, including immune cells, vasculature, stromal elements, and hypoxic conditions, and how these strategies enhance the effectiveness of current cancer immunotherapies. Additionally, we examine how intrinsic NP properties, such as size, charge, shape, and biodegradability, play critical roles in shaping their immunological outcomes.

## NPs Modulate Tumor-Associated Immune Cells

### NPs targeting T cells

T cells are central to antitumor immunity, yet their efficacy is often undermined by the hostile and immunosuppressive TME. Various components within the TME, including abnormal vasculature, stromal barriers, and immunosuppressive cytokines, restrict T cell infiltration and function [42]. This functional exhaustion limits their cytotoxic potential and enables tumor immune evasion. NP-based strategies have emerged as a promising approach to modulate T cell behavior by enhancing their infiltration, persistence, and effector function in tumors. Through precise delivery of immunomodulatory agents, NPs can both reinvigorate T cell activity and overcome suppressive cues in the TME, thereby amplifying the efficacy of cancer immunotherapy [43].

Checkpoint inhibitors have attracted substantial attention in cancer research, particularly following the approval of ipilimumab and the high-profile use of pembrolizumab. Despite their promise, these therapies are effective in only a subset of patients and can be associated with serious side effects. Consequently, nanotechnology researchers are exploring innovative strategies to modulate checkpoint pathways in tumor models, aiming to develop approaches that are both safer and more broadly effective [44]. NPs have also been employed to co-deliver ICIs alongside cytotoxic or gene-silencing agents. NP delivery systems offer the added advantage of reducing ICI dosage. For example, gold NP-based delivery of anti-PD-1 achieved equivalent therapeutic efficacy using only one-fifth of the dose required for free antibodies, thereby lowering both cost and toxicity [45]. Wang et al. [46] utilized a PD-L1 antibody to guide immunoliposomes (PD–miOXNP) encapsulating oxaliplatin and miR-130a to gastric tumors. This platform not only enabled immune checkpoint blockade but also improved intracellular delivery of therapeutic agents. Compared to free drugs or their combination, the PD–miOXNP nanoplatform significantly inhibited cell proliferation and migration in vitro and induced marked tumor regression in vivo. Similarly, Zhou and colleagues [47] engineered a poly(lactic-coglycolic acid)-polyethylene glycol (PLGA-PEG) micelle capable of co-delivering PD-L1 monoclonal antibody (mAb) and all-trans retinoic acid (ATRA) for the treatment of oral squamous cell carcinoma and dysplasia. In vivo anticancer studies indicated that ATRA-PLGA-PEG-PD-L1 was more therapeutically effective than unbound ATRA and also stimulated CD8^+^ T cell activity within the treated TME.

**Fig. 2.**
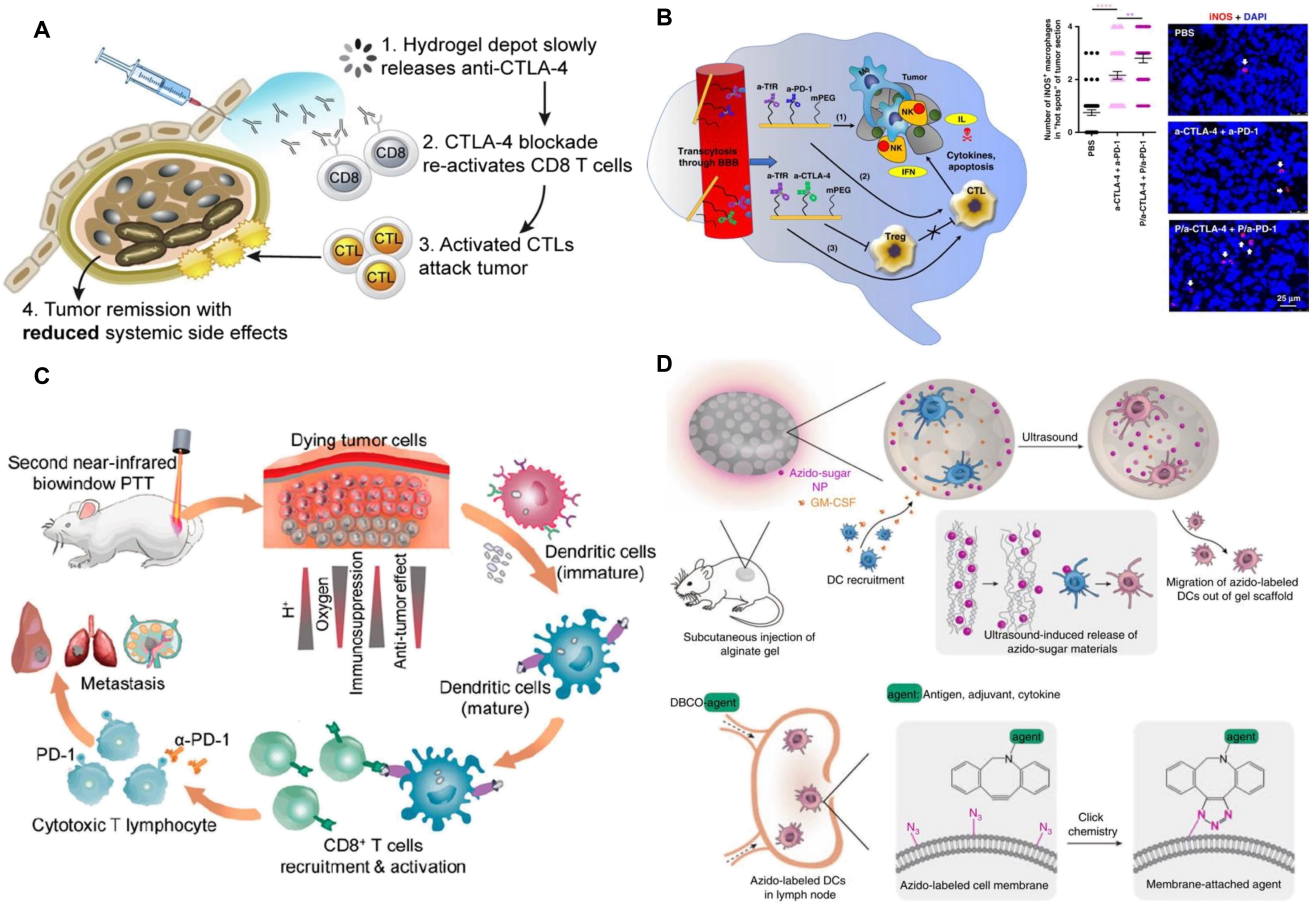
Nanomaterials remodel the TME by modulating T cells and DCs. (A) Hydrogel depot delivering anti-CTLA-4 antibodies reactivates CD8^+^ T cells, enhancing CTL responses and inducing tumor regression while minimizing systemic toxicity. Reproduced with permission from [51]. Copyright 2020, Elsevier. (B) Nano-immunoconjugates derived from poly(β-l-malic acid), linked with anti-CTLA-4 or anti-PD-1, facilitate systemic delivery across the BBB and trigger local brain antitumor immune responses. Reproduced with permission from [50]. Copyright 2019, Springer Nature. (C) Self-assembled gold NPs support NIR-II photothermal therapy, promoting deep and uniform immunogenic cell death in solid tumors and stimulating both innate and adaptive immune responses for tumor suppression and metastasis inhibition. Reproduced with permission from [69]. Copyright 2019, American Chemical Society. (D) Biomaterial-recruited DCs are metabolically tagged with azido groups in situ, allowing for their long-term tracking and targeted functional modulation. Reproduced with permission from [75]. Copyright 2020, Springer Nature.

Moreover, CTLA-4 is a pivotal immune checkpoint that negatively regulates T cell activation and plays a key role in maintaining immune homeostasis [48]. Its inhibition has emerged as a promising strategy to enhance antitumor immunity, including in aggressive cancers such as glioblastoma. Targeting CTLA-4 can stimulate cytotoxic T cell activity and remodel the TME, making it an attractive focus for novel immunotherapeutic approaches [49]. Building on this concept, Galstyan et al. [50] integrated nanotechnology with immunotherapy by developing nanoscale immunoconjugates (NICs) based on a natural biopolymer scaffold, poly(β-l-malic acid), covalently linked to anti-CTLA-4 antibodies. As illustrated in Fig. 2B, this platform was designed for systemic administration and effective penetration across the blood–brain barrier (BBB), thereby enabling localized antitumor immune responses within the brain. In a murine GL261 glioblastoma, a model NIC treatment resulted in a notable increase in cytotoxic CD8^+^ T cells, natural killer (NK) cells, and macrophages, along with a reduction in regulatory Tregs within the TME. Compared to free anti-CTLA-4 or NICs carrying only a single immune checkpoint inhibitor, the combinatorial NIC therapy significantly extended survival in glioblastoma-bearing mice. These results underscore the therapeutic potential of BBB-penetrating, tumor-targeted nanoconjugates in reprogramming both local and systemic immune responses for glioblastoma treatment.

In addition to systemic delivery approaches, localized release systems have also been explored. Leveraging thermosensitive poloxamer 407 (P407) hydrogels, Chung et al. developed a controlled-release platform for anti-CTLA-4 antibodies. The hydrogel exhibited a porous structure with vesicle sizes around 28 nm at P407 concentrations of 5% and 7.5%. The antibodies were fully incorporated into the hydrogels without loss of bioactivity. This therapeutic approach effectively inhibited tumor growth, and the sustained release from the P407 hydrogel has the potential to lower systemic antibody exposure and reduce the adverse effects commonly associated with CTLA-4 therapies (Fig. 2A) [51].

Another study introduced a novel fluorinated-coordinative-epigallocatechin gallate (EGCG) delivery system, termed FEGCG/Zn, created by incorporating fluorination and zinc ions (Zn^2+^) into EGCG. The therapeutic potential of FEGCG/Zn was evaluated based on its impact on PD-L1 expression. By modulating PD-L1 levels in tumor cells, the combination of FEGCG/Zn and siPD-L1 alleviates T cell fatigue, thereby supporting cancer immunotherapy. FEGCG/Zn not only significantly modulates PD-L1 expression but also enhances the transport of immune biomolecules by forming biomimetic nanoassemblies, offering a flexible and promising platform for cancer immunotherapy [52].

To enhance T cell infiltration into the TME, it is crucial to address TGF-β, a central regulator within the microenvironment that restricts T cell entry and dampens immune activation. Inhibition of TGF-β has been shown to enhance lymphocyte penetration and promote a more immunogenic microenvironment [53,54]. Recently, Yi et al. [55] designed a bispecific antibody targeting both TGF-β and PD-L1, termed BiTP, and demonstrated its therapeutic superiority over monotherapies in triple-negative breast cancer (TNBC). BiTP treatment resulted in reduced collagen deposition, increased CD8^+^ T cell infiltration, and elevated tumor-infiltrating lymphocytes (TILs), thereby enhancing antitumor responses.

Chimeric antigen receptor (CAR)-T cell therapy has demonstrated remarkable efficacy in treating hematologic cancers and represents a key approach in cellular immunotherapy for tumors. NPs are commonly employed for nucleic acid delivery and can be directed toward specific cell types through surface-conjugated antibodies [56]. In a recent study, Smith et al. [57] engineered CAR-T cells directly in vivo using polymeric NPs. By modifying the NPs with anti-CD3ε F(ab′)2 fragments and encapsulating plasmids encoding a murine anti-CD19 CAR along with a hyperactive transposase, the system selectively introduced CAR genes into T cells, achieving sustained tumor remission.

Additionally, immunotherapy has transformed the treatment landscape of multiple malignancies, with bispecific T cell engagers (BiTEs) emerging as one of the most potent modalities [58]. BiTEs function by simultaneously binding to the CD3 receptor on T cells and to a tumor-associated antigen (TAA) on cancer cells, thereby redirecting T cells to lyse malignant targets in a major histocompatibility complex (MHC)-independent manner. Traditional recombinant BiTEs are small proteins (~55 kDa) that undergo rapid renal clearance, necessitating continuous infusion to maintain therapeutic levels [59]. In addition, their systemic distribution increases the risk of cytokine release syndrome and off-tumor toxicity. By embedding BiTEs (NP bispecific T cell engagers) within NPs, several advantages can be achieved, including improved pharmacokinetics, targeted delivery, multifunctionality, and the ability to overcome stromal barriers [60]. One representative example is the recent work in multiple myeloma where liposome-based nanoBiTEs and “multi-antigen” BiTEs (or nanoMuTEs) were constructed by chemical conjugation of anti-CD3 together with antibodies against one or more tumor antigens (e.g. BCMA, CS1, and CD38) on the NP surface. The nanoMuTEs targeting multiple antigens showed superior efficacy in vitro and in vivo compared with nanoBiTEs targeting a single antigen. Importantly, these nanosystems exhibited a much longer half-life (~60 h), enabling less frequent dosing [61]. In another study, Cheng et al. [62] loaded a chemotherapeutic agent (ganetespib) into PEGylated nanocarriers, and BiTEs (CD3/PD-L1) are noncovalently attached (via anti-mPEG linkers) to the surface. These BiTE-decorated nanocarriers (BiTEs-GSP-NCs) enhance cellular uptake, increase T cell-mediated cytotoxicity, and improve tumor growth inhibition in vivo, compared to nontargeted or free BiTE/drug components. Similarly, Alhallak et al. [61] developed a nanoBiTEs, consisting of liposomes functionalized with 2 mAbs: anti-CD3 to engage T cells and anti-CD20 to target malignant B cells. Their therapeutic efficacy was evaluated in a 3D culture model of Waldenstrom macroglobulinemia, a subtype of non-Hodgkin lymphoma. Treatment with CD20/CD3 nanoBiTEs induced significant cytotoxicity, resulting in 60% to 70% cell death, whereas control formulations using nonspecific isotype antibodies showed no measurable effect. In vivo, mice treated with isotype/CD3 nanoBiTEs experienced rapid tumor progression and succumbed within 21 d. In contrast, mice receiving the CD20/CD3 formulation exhibited a marked delay in tumor growth by days 14 and 21, with near-complete tumor eradication observed by day 35. Building on this strategy, the researchers also developed multispecific NPs (nanoMuTEs) by conjugating multiple mAbs targeting various cancer antigens. In both in vitro and in vivo models of multiple myeloma, nanoMuTEs demonstrated superior antitumor efficacy compared to single-targeted nanoBiTEs, underscoring the potential of multispecific NP platforms to enhance T cell-mediated immunotherapy.

Together, these advancements underscore the multifaceted potential of NP-based platforms in enhancing T cell-mediated cancer immunotherapy. By improving targeting precision, enabling the co-delivery of synergistic agents, and facilitating transport across challenging barriers such as the BBB, these systems offer notable advantages over conventional immunotherapeutic approaches. Moreover, multifunctional nanocarriers provide avenues to modulate the immunosuppressive TME, boost T cell activation, and mitigate immune exhaustion.

### NPs targeting DCs

Dendritic cells (DCs) act as a vital bridge between the innate and adaptive immune systems by presenting antigens and activating naïve T cells. As such, strategies that enhance antigen delivery to and presentation by DCs hold considerable promise in cancer immunotherapy [63]. NPs have emerged as versatile tools for modulating DC function by enabling targeted antigen delivery, promoting DC maturation, and supporting efficient T cell priming [64,65].

One example is the development of ultra-pH-sensitive polymeric NPs designed to deliver the model antigen ovalbumin (OVA) without the need for additional adjuvants. In a study by Luo et al. [66], these ~30-nm NPs were constructed with a cyclic 7-membered ring structure that conferred strong immunogenic properties by engaging the stimulator of interferon genes (STING) and activating type I interferon signaling pathways. This activation substantially enhanced DC maturation, enabling efficient cross-presentation of antigens. The mature DCs subsequently promoted the proliferation and functional activation of OVA-specific CTLs. In preclinical models, this NP-based vaccine demonstrated robust immunogenicity and effectively suppressed tumor growth across multiple cancer types, including B16F10 melanoma, MC-38 colon carcinoma, and human papillomavirus (HPV)-associated cancers.

Another promising approach leverages the immunogenic effects of phototherapy. Studies have shown that TAAs released from dying tumor cells following phototherapy can act as endogenous adjuvants, promoting DC maturation, stimulating cytokine secretion, and enhancing T cell responses [67]. This suggests that TAAs generated during phototherapy-induced cell death may function as adjuvants, triggering immune activation and effectively serving as a form of “automatic vaccination” [68,69]. As shown in Fig. 2C, immunogenic cell death (ICD) induced by fluid liposome-coated gold NPs activated both innate and adaptive immunity. This response facilitated the full maturation of DCs and supported the proliferation of NK cells as well as interferon-γ (IFN-γ)-producing CD4^+^ and CD8^+^ T cells. Although these NPs did not increase the total number of DCs, they substantially enhanced DC maturation at 48 and 72 h following photothermal therapy (PTT) [69]. Direct targeting of DCs has also been achieved using surface markers. For example, subcutaneous administration of 1-μm particles conjugated with anti-CD205 antibodies substantially increased uptake by CD205^+^ antigen-presenting cells (APCs) and enhanced their trafficking to draining lymph nodes [70]. Similarly, lentiviral vectors functionalized to target DC-specific intercellular adhesion molecule-3-grabbing non-integrin (DC-SIGN) exhibited a 10-fold increase in lymph node accumulation compared to untargeted vectors, further supporting the strategy of DC-specific delivery [71].

The spatial arrangement and density of targeting ligands on NPs also play critical roles in determining immunological outcomes. For instance, PLGA NPs targeting DEC-205 showed that increased ligand density correlated with enhanced cellular immunity [72]. In another study, Wilson et al. [73] developed a synthetic glyco-adjuvant, p(Man-TLR7), designed to target both Toll-like receptor 7 (TLR7) and mannose receptors on DCs. In order to release chemically unaltered antigen to present to T cells, the p(Man-TLR7) was next connected with the OVA using a copper-free click technique. The subunit nanovaccines’ immunogenicity was improved by this synthetic glyco adjuvant, which also served as a basis for vaccine development. In a separate study, Kim et al. [74] introduced a TLR7/8 dual-ligand nanovaccine, termed 522NPs, based on a PLGA carrier. Upon administration, these NPs trafficked to draining lymph nodes, where they robustly activated DCs and elicited CTL responses, further underscoring the potential of NP-mediated DC targeting in cancer immunotherapy.

To further enhance DC targeting and activation, Wang et al. [75] employed a combined strategy involving metabolic glycoengineering and click chemistry. In this system, DCs were recruited into alginate gels containing granulocyte-macrophage colony-stimulating factor (GM-CSF) and azido-sugar-loaded NPs. Once metabolically labeled with azido groups, these DCs were selectively targeted using dibenzocyclooctyne (DBCO)-conjugated agents for the delivery of antigens and immunomodulators. This approach promoted DC maturation and cytokine production, leading to enhanced antitumor immune responses (Fig. 2D). In a related study, Xu et al. [76] developed a nanovaccine based on a polyamidoamine (PAMAM) dendrimer functionalized with guanidinobenzoic acid (DGBA) to improve antigen delivery and cross-presentation by DCs. The system incorporated OVA as a model antigen and unmethylated CpG as an adjuvant. The resulting DGBA–OVA–CpG nanovaccine elicited strong antigen-specific cellular immune responses and demonstrated substantial prophylactic efficacy against B16-OVA melanoma. When combined with PD-1 checkpoint blockade, this treatment produced notable therapeutic effects, increasing the mouse survival rate to nearly 38% within 2 months in melanoma-bearing models.

Cancer cell membrane-coated NPs represent another innovative class of DC-targeting systems. Gou et al. [77] designed ~150-nm PLGA NPs coated with tumor cell membranes and functionalized with peptides targeting Clec9a, a DC-specific receptor. These particles co-delivered tumor antigens along with the STING agonist 2′3′-cyclic guanosine monophosphate-adenosine monophosphate (cGAMP) to DCs, stimulating type I interferon responses and enhancing antigen cross-presentation. The platform substantially increased polyfunctional CD8^+^ T cell responses and exhibited synergistic therapeutic effects when combined with radiotherapy in B16F10-OVA and 4T1 tumor models.

Targeting peripheral DCs via surface markers such as CD40, MHC-II, and C-type lectin receptors (e.g., CD205 and CD206) requires high specificity and rapid internalization into antigen-processing compartments [78]. To address this, Li et al. [79] developed a magnetically guided nanovaccine by coating CpG-loaded Fe_3_O_4_ magnetic nanoclusters with cancer cell membranes and anti-CD205 antibodies. These NPs accumulated in lymph nodes via magnetic targeting and prolonged antigen retention, thereby enhancing DC uptake and cross-presentation through MHC-I pathways. The cancer cell membrane coating also provided a broad repertoire of tumor antigens, supporting a multifaceted antitumor immune response.

Beyond the DCs residing in the TME, the maturation of DCs within lymph nodes is critically important for effective cancer immunotherapy. Lymph-targeting NPs can play a pivotal role in shaping antitumor immunity by promoting the trafficking of TAAs to lymph nodes, thereby facilitating cross-presentation by DCs and enhancing T cell priming and activation [80]. Several studies have demonstrated that NPs with optimized size (10 to 100 nm), surface charge, and lymphatic affinity can drain efficiently from the tumor site or injection site into the lymphatics, where they are preferentially taken up by APCs. NP size, surface charge, and composition are decisive factors that govern lymph node delivery. Studies have shown that NPs in the 10- to 100-nm range can efficiently drain from peripheral injection sites into lymphatic capillaries and accumulate within lymph nodes, whereas larger particles (>200 nm) are often retained at the injection site [81]. Ultra-small NPs (~25 nm) have demonstrated efficient transport through lymphatic capillaries and enhanced accumulation in lymph nodes, leading to improved DC activation and T cell priming [82]. Beyond passive delivery, surface modification of NPs with targeting ligands further improves lymph node-specific DC engagement. Kranz et al. [83] have utilized liposomes conjugated with DC-targeting peptides to deliver tumor antigens directly to lymph node-resident DCs, thereby improving antigen cross-presentation and T cell priming. The findings demonstrated that liposomes protect RNA from degradation by extracellular ribonucleases and promote its efficient uptake and antigen expression within DCs and macrophages across multiple lymphoid tissues, ultimately inducing strong effector and memory T cell responses and mediating potent IFN-α-dependent rejection of progressive tumors. Importantly, NP-mediated regulation of the lymphatic system offers a strategy to overcome one of the major hurdles in cancer immunotherapy—the immunosuppressive TME. By diverting tumor antigens away from local immune suppression toward the lymphatic system, lymph node-targeting NPs amplify the likelihood of productive DC maturation and cytotoxic T cell expansion [84]. Taken together, these findings underscore the potential of lymph node-targeting NPs to regulate the immune response at its origin. By promoting antigen trafficking to lymph nodes, enhancing DC cross-presentation, and facilitating T cell priming, such systems bridge the gap between antigen delivery and adaptive immunity [85].

Collectively, these approaches underscore the multifaceted role of nanotechnology in enhancing DC targeting and function, ultimately boosting antigen-specific T cell immunity. The advancement of targeted nanovaccines offers substantial promise for optimizing immune responses against solid tumors, especially when integrated with existing immunotherapeutic strategies.

### NPs target NK cells

**Fig. 3.**
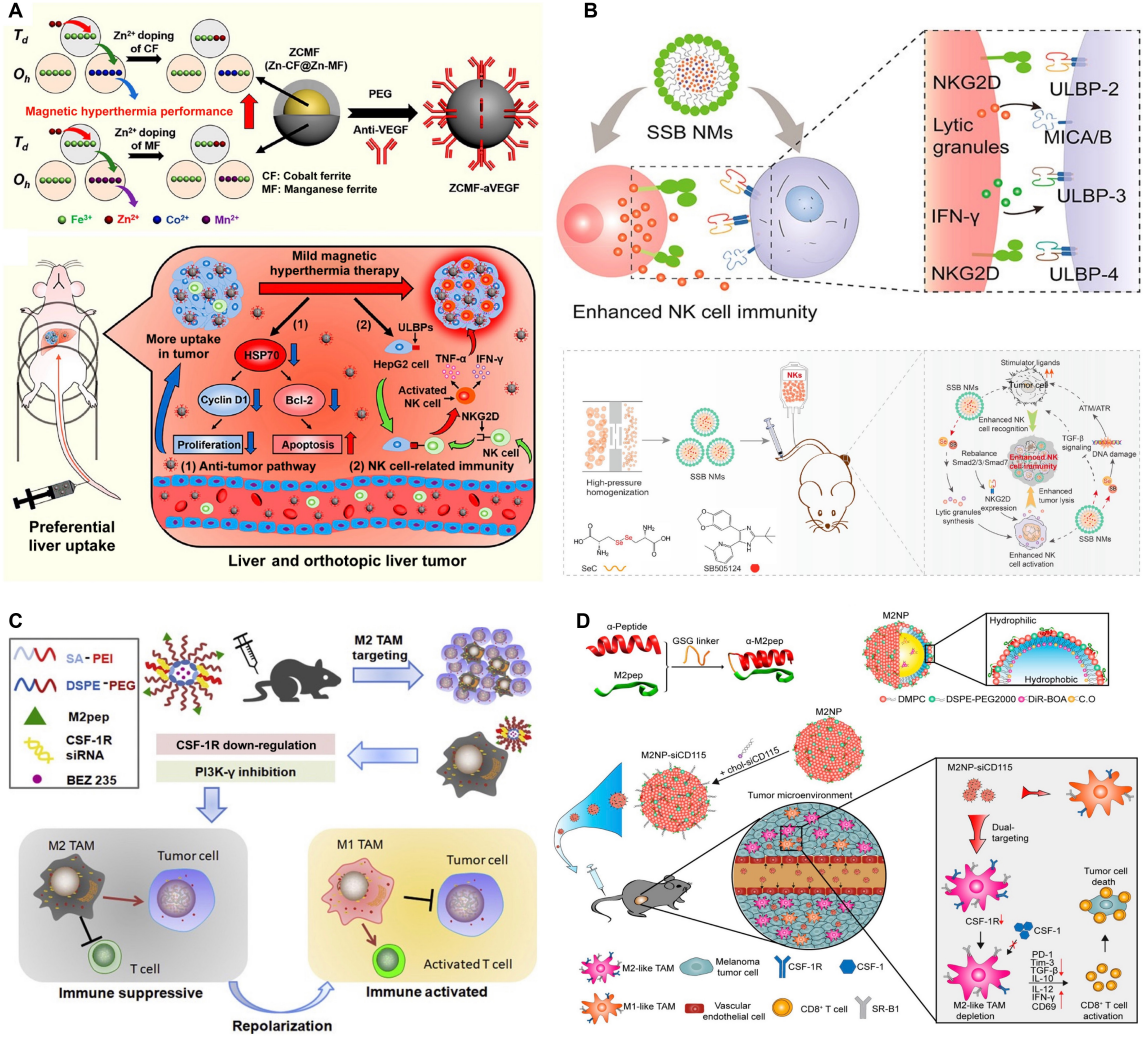
Nanomaterials for enhancing innate immunity through modulation of NK cells and TAMs. (A) Core–shell superparamagnetic NPs with exchange-coupled magnetism enable efficient and controllable mild magnetic hyperthermia, offering a promising therapeutic strategy for orthotopic liver cancer. Reproduced with permission from [87]. Copyright 2021, American Chemical Society. (B) A NE co-delivering a TGF-β inhibitor and SeC enhances anticancer efficacy by promoting NK cell-mediated immune responses. Reproduced with permission from [88]. Copyright 2020, American Chemical Society. (C) An M2 macrophage-targeted nanomicelle, composed of M2pep-1,2-distearoyl-sn-glycero-3-phosphoethanolamine (DSPE)-PEG and poly(ethyleneimine)-stearic acid (PEI-SA), was designed to co-deliver the PI3K-γ inhibitor NVP-BEZ235 and CSF-1R siRNA, enabling precise reprogramming of the tumor immune microenvironment and enhancing therapeutic outcomes in pancreatic cancer. Reproduced with permission from [109]. Copyright 2020, Elsevier. (D) M2-like TAM-targeting NPs (M2NPs), functionalized with an α-peptide and M2pep and loaded with anti-CSF-1R siRNA, selectively deplete immunosuppressive macrophages in melanoma by inhibiting their survival signaling. Reproduced with permission from [110]. Copyright 2017, American Chemical Society.

NK cells play a crucial role in innate immunity and are increasingly recognized for their potential in cancer immunotherapy. Nanotechnology presents novel strategies to enhance NK cell activity by modulating the TME, boosting cytotoxic function, and enabling targeted gene delivery [86]. As illustrated in Fig. 3A, Pan et al. [87] developed a supermagnetic NP, Zn-CoFe_2_O_4_@Zn-MnFe_2_O_4_, for use in magnetic hyperthermia therapy (MHT). Mild MHT substantially up-regulated UL16-binding proteins (ULBPs), ligands of the NKG2D receptor, on liver cancer cells. This led to enhanced NK cell activation and increased secretion of tumor necrosis factor-α (TNF-α) and IFN-γ, ultimately suppressing tumor growth in both xenograft and orthotopic models. In a similar approach, a nanoemulsion (NE) system was designed to co-deliver a TGF-β inhibitor and selenocysteine (SeC) for improved anticancer efficacy. This formulation nearly doubled the cytotoxic activity of NK92 cells. Mechanistically, the enhanced immune response was driven by activation of the NKG2D/NKG2D ligand signaling axis and engagement of the DNA damage response. The NE also inhibited the TGF-β/TGF-βRI/Smad2/3 pathway, resulting in elevated NKG2D ligand expression on tumor cells and increased NKG2D levels on NK92 cells. Additionally, SeC promoted γδ T cell cytotoxicity by up-regulating NKG2D expression while down-regulating PD-1. Collectively, these effects highlight the promise of NE-based co-delivery platforms to amplify NK and γδ T cell responses by approximately 2-fold, achieving a tumor suppression rate nearing 80%, and offering a compelling strategy for adoptive cancer immunotherapy (Fig. 3B) [88].

To relieve immunosuppression, Adjei et al. [89] developed a manganese dioxide (MnO_2_) NP system to deliver small interfering RNA (siRNA) targeting transforming growth factor β receptor-2 (TGFBR2), a known inhibitor of NK cell function. These NPs, loaded with TGFBR2 siRNA, achieved a 90% reduction in TGFBR2 receptor expression and shielded NK cells from immunosuppressive signaling. Consequently, TGFBR2 suppression enhanced the immune responsiveness of the TME by activating NK cells, leading to a notable 30% increase in IFN-γ production. This indicates that such NPs can strengthen the anticancer effects of NK cells by inhibiting TGFBR2 and boosting IFN-γ expression.

Exosome-based delivery systems have also gained attention. Neviani et al. [90] utilized NK cell-derived exosomes modified with miRNA-186. These engineered exosomes showed substantial cytotoxicity against neuroblastoma cell lines with amplified MYCN. Moreover, the cytotoxic effect was positively correlated with miRNA-186 expression levels. The exosomes down-regulated TGF-β, a key player in tumor immune evasion, as demonstrated in vitro. The results suggest that NK cell-derived exosomes carrying miRNA-186 may serve as a promising therapeutic tool to enhance NK cell-mediated cytotoxicity—up-regulating perforin by approximately 9-fold and granzyme B by about 3-fold, while impairing tumor cell immune escape mechanisms.

NPs conjugated with bispecific antibodies have been employed to physically link NK cells to tumor cells [91]. These bispecific antibodies act as molecular bridges, facilitating direct interactions between NK cells and cancer cells, thereby promoting NK cell-mediated cytotoxicity. A novel approach utilizes PEGylated hollow mesoporous ruthenium NPs, which are conjugated with bispecific antibodies (SS-Fc). The SS-Fc molecule was specifically engineered with one arm targeting TAAs, namely, anti-carcinoembryonic antigen (CEA), and the other arm engaging CD-16, an activating receptor on NK cells. This dual recognition enabled effective crosslinking of NK cells to tumor cells, leading to potent cytotoxic responses against tumors overexpressing the target antigens [92].

CAR-modified NK cells (CAR-NK) are emerging as a safer and potentially more versatile alternative to CAR-T cells. Upon CAR modification, NK cells gain an enhanced ability to recognize and eliminate cancer cells through multiple mechanisms, including the release of cytotoxic agents and the induction of programmed cell death [93]. In contrast to CAR-T cell therapy, which is associated with cytokine storms in up to 30% of patients, CAR-NK cell therapy has shown no reported cases of this adverse effect, reflecting the immune-regulatory nature of NK cells [94]. The use of NPs to deliver CAR genes into patient-derived NK cells offers a promising strategy for developing CAR-NK-based therapies. To this end, Kim et al. [95] synthesized a cationic polymer labeled with a near-infrared (NIR) dye and coated with polydopamine (PDA), referred to as MF-NP. This polymer was used to deliver plasmid DNA encoding epidermal growth factor receptor (EGFR)-CARs to NK cells. MF-NPs exhibited high cytocompatibility and efficient gene transfection, leading to robust expression of the target protein. Application of MF-NP resulted in a 60% increase in EGFR-CAR expression on the surface of NK-92MI cells, thereby enhancing their antitumor immune response. Additionally, MF-NP enabled in vivo tracking of NK cells through both magnetic resonance and fluorescence imaging.

Siegler et al. [96] utilized NK cells genetically modified with CARs to target crosslinked multilamellar liposomal vesicles (cMLVs) loaded with paclitaxel (PTX). The cationic multilamellar vesicles were functionalized with maleimide head groups, which react with sulfhydryl groups. This chemical modification enabled stable binding of cMLVs to the surface of NK cells, which naturally express abundant sulfhydryl groups. In vivo studies showed that mice treated with CAR-NK cells conjugated to cMLV (PTX) combining NK cell-mediated immunotherapy with PTX-mediated chemotherapy exhibited significantly greater tumor volume reduction compared to those receiving either cMLV (PTX) or CAR-NK cells alone.

Gene delivery using chitosan NPs encapsulating IL-21 and NKG2D plasmids has been shown to further enhance NK and CD8^+^ T cell activity. In vitro studies demonstrated that loading chitosan NPs with IL-21 and NKG2D genes via plasmids activated both NK cells and cytotoxic T cells. Tan et al. [97] reported increased tumor accumulation of the NPs, attributed to the EPR effect, along with greater lymphocyte infiltration within the TME. Their findings revealed significantly reduced tumor volumes and prolonged survival—up to nearly 40 d compared to 22 d in the control group—in mice bearing CT-26 tumors, aligning with the in vitro results. These outcomes indicate that effective activation of NK cells and enhancement of antitumor responses can be achieved through IL-21 delivery via chitosan-based NPs.

Zhuo et al. [98] developed a method using polyethylene glycol (PEG)-modified dendrimer-entrapped gold NPs to target breast cancer cells via NK cells transfected with the human ferritin heavy chain (hFTH1) gene. This strategy demonstrated effective cancer cell destruction. PEG-modified PAMAM dendrimers were used as templates for gold NP entrapment to facilitate hFTH1 transfection into NK cells. The resulting NPs exhibited excellent imaging properties and high transfection efficiency, achieving a rate of 80.2% at an N/P ratio of 5:1. The study showed that the NPs effectively guided NK-92 cells toward tumor cells, enabling efficient gene delivery with minimal impact on NK cell function. This work establishes a novel research framework for NK cell-based immunotherapy, with the ultimate goal of advancing and personalizing immunotherapeutic strategies for breast cancer. Collectively, these studies underscore the versatility of nanotechnology in modulating NK cell behavior for cancer immunotherapy, presenting promising strategies to improve tumor targeting, enable gene editing, and enhance immune activation while minimizing systemic toxicity.

### NPs target TAMs

TAMs represent a predominant immune cell population within the TME, where they generally adopt an M2-like, immunosuppressive phenotype. These M2 macrophages facilitate tumor progression by promoting angiogenesis, suppressing cytotoxic immune responses, and enabling metastasis [99]. As a result, targeting or reprogramming M2-like TAMs has emerged as a compelling strategy in cancer immunotherapy. NP-based delivery systems offer several advantages for manipulating TAM behavior, including enhanced targeting specificity, co-delivery potential, and reduced systemic toxicity [100].

In a notable study, Zhang et al. [101] developed ultrasmall copper-based NPs (Cu@CuO_x_) functionalized with a peptide targeting C–C chemokine receptor type 2 (CCR2), which is overexpressed on protumorigenic TAMs. These multifunctional NPs served dual functions: enabling sensitive positron emission tomography imaging via intrinsic ^64^Cu radiolabeling and delivering the chemotherapeutic agent gemcitabine. The nanocarriers demonstrated favorable pharmacokinetics, efficient renal clearance, and low in vivo toxicity. When administered systemically, gemcitabine-loaded Cu@CuO_x_ NPs markedly inhibited tumor growth in pancreatic ductal adenocarcinoma (PDAC) across both syngeneic xenograft and autochthonous genetically engineered mouse models. Their precision in both detection and therapy underscores their promise as a platform for image-guided treatment and clinical translation. Another innovative approach involves CXCL12-encapsulated PLGA/pluronic NPs designed to disrupt the CXCL12/CXCR4 signaling axis—a key driver of tumor cell migration and metastasis. These NPs function as antagonists, blocking metastatic spread while potentially attenuating the tumor-promoting role of TAMs [102].

MicroRNAs (miRNAs) have also been utilized to reprogram TAM phenotypes. miR-125b, a miRNA known to promote M1 macrophage polarization, was delivered using CD44-targeted hyaluronic acid-poly(ethylenimine) (HA-PEI) NPs in a non-small cell lung cancer (NSCLC) model. This strategy increased inducible nitric oxide synthase (iNOS) expression and decreased arginase-1 activity, markers of M1 and M2 macrophages, respectively, effectively shifting the TAM population toward a proinflammatory, antitumor phenotype [103]. Similarly, Hu et al. [104] transfected miR-125b into macrophages using anionic magnetic NPs in a 4T1 orthotopic breast cancer model, achieving comparable macrophage repolarization and therapeutic effects.

Selective targeting of the mannose receptor (CD206), a surface marker highly expressed on M2 macrophages, has also been explored. In one study, Fe_3_O_4_-based PLGA NPs were surface-functionalized with anti-CD206 mAbs to selectively bind M2 TAMs. These particles, measuring 260 to 295 nm in size, induced the production of reactive oxygen species (ROS) and up-regulated proinflammatory mediators such as TNF-α, iNOS, and IL-1β in vitro. In vivo, CD206-targeted NPs increased CD86 expression, an M1 macrophage marker, demonstrating effective TAM reprogramming via iron oxide-induced oxidative stress [105].

Ferumoxytol, a U.S. Food and Drug Administration (FDA)-approved iron oxide NP, has also demonstrated immunomodulatory capabilities [106]. Zanganeh et al. [107] reported that ferumoxytol NPs exerted a therapeutic effect on the progression of early-stage breast tumors and lung cancer metastases in the liver and lungs. Exposure to ferumoxytol enhanced macrophages expression of mRNAs associated with proinflammatory Th1-type responses. This was accompanied by an increase in M1 macrophages and suppression of tumor growth within the TME.

An innovative strategy to enhance macrophage phagocytic activity involved the development of soluble, multivalent self-peptides designed to inhibit signal regulatory protein α (SIRPα), a key negative regulator of phagocytosis. Jalil et al. [108] engineered bivalent and tetravalent nano-self peptides that effectively disrupted the CD47–SIRPα interaction, enhanced antibody-mediated phagocytosis, and suppressed downstream phosphotyrosine signaling. This approach offers a promising alternative for boosting innate immune responses against tumor cells.

As illustrated in Fig. 3C, Li et al. [109] developed a targeted nanomicelle system to reprogram TAMs and augment antitumor immunity in pancreatic cancer. By functionalizing mixed micelles with the M2 TAM-specific peptide M2pep, they co-delivered the phosphatidylinositol 3-kinase-γ (PI3K-γ) inhibitor NVP-BEZ235 and CSF-1R siRNA to selectively target M2-polarized TAMs. This dual-delivery platform demonstrated enhanced targeting efficiency both in vitro and in vivo. Compared to single-pathway inhibition, the combined blockade of PI3K-γ and CSF-1R more effectively shifted macrophage polarization from the immunosuppressive M2 phenotype to the proinflammatory M1 state while concurrently reducing MDSC infiltration. As a result, the treatment substantially enhanced antitumor immune responses and suppressed pancreatic tumor growth. This work underscores the therapeutic potential of synergistic TAM reprogramming through co-delivery of RNA and small-molecule inhibitors to remodel the tumor immune microenvironment. In a related study, Qian et al. [110] designed dual-targeting NPs (M2NPs) to selectively deplete M2-like TAMs in melanoma. These NPs were engineered using a fusion peptide comprising an SR-B1-targeting α-peptide and M2pep, which binds specifically to M2 macrophages. The incorporation of siRNA against colony stimulating factor-1 receptor (CSF-1R), a key survival factor for M2-like TAMs, enabled targeted immunotherapeutic depletion of these cells. The fusion peptide improved specificity and uptake in M2-like TAMs while sparing resident macrophages in nontumor tissues. Treatment with M2NPs resulted in nearly 50% TAM depletion, an 87% reduction in tumor burden, and improved survival in mouse models (Fig. 3D).

Overall, these studies underscore the versatility of NP-based platforms in reprogramming or depleting TAMs to reshape the immunosuppressive TME. By enabling the targeted delivery of small molecules, RNA therapeutics, or immunomodulatory agents, nanomedicine shows substantial potential to enhance the efficacy of cancer immunotherapies by overcoming macrophage-driven resistance mechanisms.

## NPs Target CAF

CAFs are key components of the TME and play vital roles in promoting tumor progression. Originating from local stromal fibroblasts, mesenchymal stem cells, or other progenitor sources, fibroblasts typically remain quiescent under normal physiological conditions [111]. However, in response to tumor-derived signals such as TGF-β1, they become activated and differentiate into CAFs. Once activated, CAFs contribute to ECM remodeling, promote angiogenesis, support tumor cell proliferation and metastasis, and modulate immune responses within the TME [112]. The tumor stroma comprises fibroblasts that contribute to tumor progression through reciprocal communication with neoplastic cells. As key cellular components of the TME, CAFs display a heterogeneous makeup originating from diverse sources, including local fibroblasts and mesenchymal stem cells [113]. Under normal conditions, fibroblasts typically remain quiescent in healthy tissues and during the early stages of tumor development [114]. However, as tumors advance, fibroblasts undergo a series of physiological and biochemical changes, leading to their activation and differentiation into CAFs. CAFs are instrumental in several essential processes, including ECM remodeling, modulation of the immune response, promotion of angiogenesis, and enhancement of cancer cell proliferation and metastatic spread, as extensively described in earlier reviews [115].

One promising strategy for targeting CAFs involves the use of cerium oxide NPs (nanoceria), which exhibit both prooxidant and antioxidant properties depending on their oxidation state [1]. Alili et al. [116] synthesized nanoceria with the aim of directly damaging tumor cells and inhibiting myofibroblast formation. In the presence of Ce4^+^, nanoceria acts as a prooxidant, increasing intracellular ROS levels and selectively targeting tumor cells. Importantly, the concentrations of nanoceria remain nontoxic to normal stromal cells while exerting cytotoxic effects on tumor cells. Nanoceria possesses unique features whereby, at nontoxic doses, it blocks the TGF-β1-and ROS-induced transformation of fibroblasts into myofibroblasts, thereby enabling selective delivery to CAFs. Treatment with nanoceria led to a reduction in α-smooth muscle actin (α-SMA) expression, indicating suppression of the fibroblast-to-myofibroblast transition. Further evaluation of its effect on tumor invasiveness revealed a significant 70% decrease in invasive potential in the nanoceria-treated group. These results suggest that nanoceria plays a dual role in cancer immunotherapy. In contrast, the therapeutic potential of Fe_3_O_4_ NPs, previously noted for their cytotoxicity in mammalian cells, was also examined. Researchers evaluated their impact on both tumor and normal squamous cells. However, unlike nanoceria, Fe_3_O_4_ NPs were found to be less suitable for inclusion in cancer-targeting therapeutic strategies [117].

**Fig. 4.**
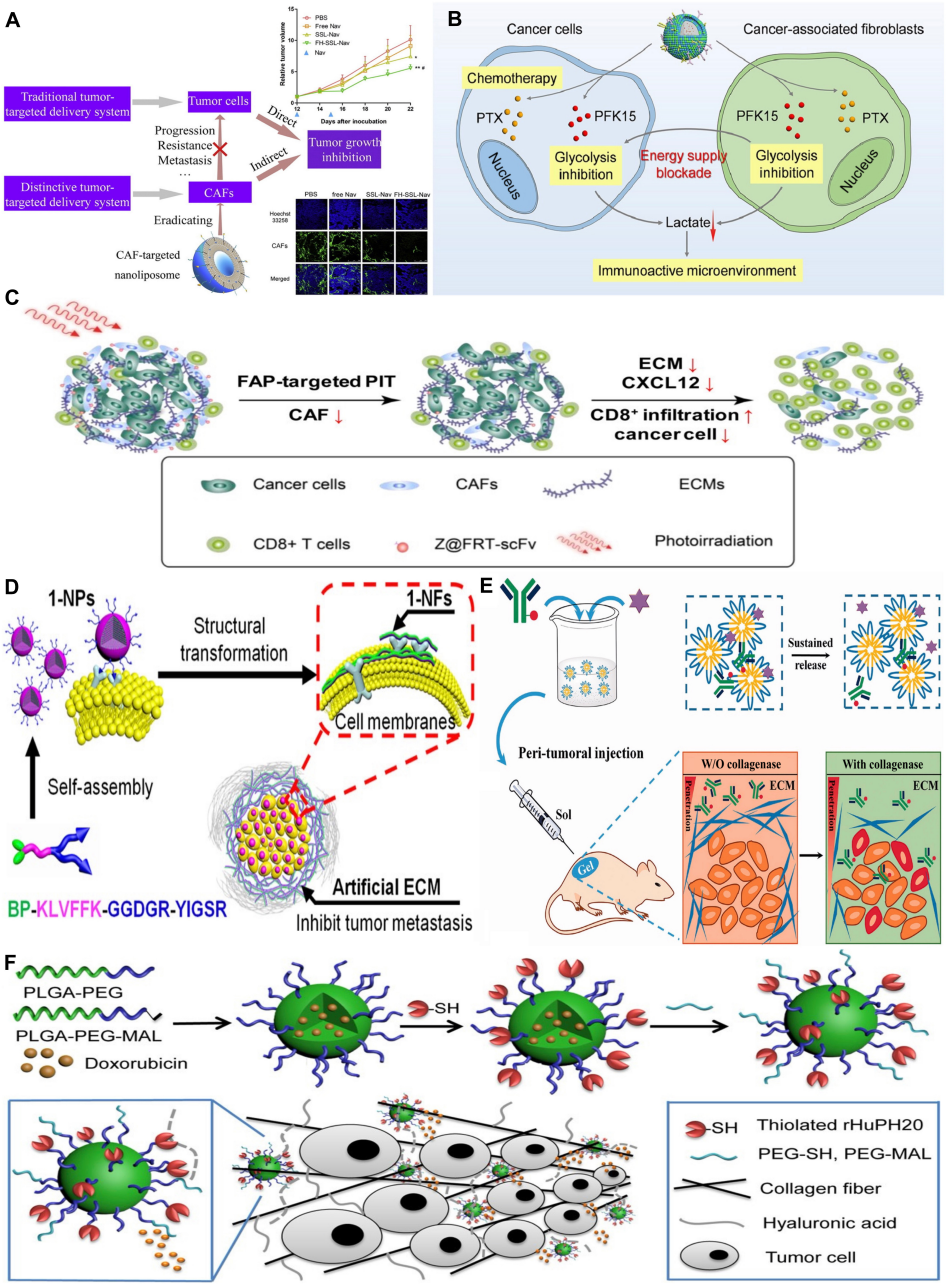
NPs targeting CAFs and ECM to remodel the TME and enhance therapeutic efficacy. (A) FH peptide-modified nanoliposomes (FH-SSL-Nav), targeting tenascin C on CAFs, facilitate the selective delivery of Nav to the tumor stroma, offering a promising strategy for cancer therapy. Reproduced with permission from [119]. Copyright 2016, Elsevier. (B) A dual-targeting biomimetic nanocarrier, coated with hybrid membranes derived from cancer cells and CAFs, was developed to co-deliver PTX and PFK15, thereby disrupting stromal metabolic support and enhancing antitumor efficacy. Reproduced with permission from [124]. Copyright 2022, Elsevier. (C) A ferritin-based NP photoimmunotherapy (nano-PIT) platform, functionalized with a FAP-specific scFv, allows for the selective elimination of CAFs upon photoirradiation while minimizing damage to healthy tissues through spatially confined activation. Reproduced with permission from [122]. Copyright 2017, American Chemical Society. (D) An artificial ECM is constructed from a transformable laminin-mimic peptide 1 that self-assembles into 1-NPs and subsequently transforms into membrane-associated nanofibrils (1-NFs), forming a biomimetic ECM barrier that inhibits tumor invasion and metastasis. Reproduced with permission from [140]. Copyright 2017, American Chemical Society. (E) A thermosensitive PLGA-PEG-PLGA hydrogel co-delivering trastuzumab and collagenase (Col/Tra/Gel) via peritumoral injection reduces collagen density and promotes tumor cell apoptosis, thereby enhancing therapeutic efficacy while minimizing toxicity. Reproduced with permission from [150]. Copyright 2018, Taylor & Francis. (F) PLGA-PEG NPs surface-modified with recombinant human hyaluronidase PH20 (rHuPH20) and shielded by a low-density PEG layer improve tumor penetration by degrading HA, enabling effective DOX delivery and suppression of aggressive 4T1 tumor growth at low drug doses. Reproduced with permission from [145]. Copyright 2016, American Chemical Society.

Another approach involves employing nanocarriers to deliver cytotoxic drugs directly to CAFs. Ji et al. [118] developed NPs incorporating a cleavable amphiphilic peptide (CAP), which is specifically cleaved by fibroblast activation protein (FAP) expressed on fibroblasts. In aqueous environments, CAP monomers readily self-assemble due to their amphiphilic properties. However, in the TME, where FAP is present on CAFs, CAP rapidly disassembles, releasing chemotherapeutic agents and selectively inducing apoptosis in CAFs. This nanostructure, notable for its rapid enzymatic response, has the potential to enhance tissue penetration and function as an efficient drug delivery platform. In another study, researchers formulated a CAF-targeting nanoliposome loaded with the cytotoxic drug navitoclax. As shown in Fig. 4A, this nanoliposome was further conjugated with peptide FH, which has a high affinity for tenascin C, a protein predominantly expressed on CAFs. Navitoclax selectively induces apoptosis in CAFs. Following treatment, the CAF population dropped to 18%, compared to 77% in the control group [119]. However, since specific CAF subtypes may exert antitumor effects at certain stages of cancer progression, their complete elimination may not represent an ideal therapeutic strategy [1].

Moreover, several studies have combined CAF-targeted therapy with photodynamic therapy (PDT), identifying FAP, which is highly expressed on CAFs, as a promising target. As a result, both an anti-FAP antibody and its humanized version, sibrotuzumab, were evaluated clinically [120]. However, following initial trials, researchers found that FAP expression in normal tissues could not be overlooked, leading to serious side effects from sibrotuzumab treatment [121]. Therefore, a more precise CAF-targeted delivery strategy is essential. Zhen et al. [122] tackled this by developing ferritin NPs functionalized with FAP-specific single-chain variable fragments (scFv) and photosensitizers. Upon irradiation, these NPs selectively eradicated CAFs, boosted T cell infiltration, and enhanced the delivery and effectiveness of other therapies (Fig. 4C).

Although CAFs have been widely recognized as barriers to effective antitumor therapies, recent studies reveal that, under certain conditions, specific CAF subsets may exhibit tumor-suppressive functions. Similar to TAMs, recent findings suggest that quiescent fibroblasts can differentiate into either cancer-inhibiting (F1 subtype) or cancer-promoting (F2 subtype) CAFs, depending on the tumorigenesis stage. Notably, the same signaling pathway may perform contrasting roles at different phases of tumor progression. As such, a targeted modulation strategy, rather than total CAF depletion, appears to be a more prudent and effective approach [123].

Inhibiting the metabolic support provided by CAFs offers a promising approach to cancer treatment. As illustrated in Fig. 4B, Zang et al. [124] fused the membranes of 4T1 cancer cells with those of activated NIH373 fibroblasts, creating a hybrid cell membrane designed to selectively target cancer cells and CAFs. Solid lipid NPs loaded with PTX, a chemotherapeutic agent, and PFK15, a glycolysis inhibitor, were then coated with these hybrid membranes. This innovative strategy aimed to suppress glycolysis, disrupt cellular energy production, and block the transfer of glycolytic metabolites from CAFs to cancer cells. The resulting inhibition of glycolysis led to reduced lactate production, which in turn down-regulated Tregs, reprogrammed M2 macrophages into the M1 phenotype, and increased the proportions of both CD8^+^ and CD4^+^ T cells.

A key limitation of many nanomedicine approaches in oncology is the reliance on a single targeted ligand to direct NPs toward one specific cell population within the TME. While such strategies can improve delivery compared to passive targeting, they may fail to account for the heterogeneity and complexity of the TME, which is composed of diverse stromal and immune populations that collectively sustain tumor progression [125]. To overcome these challenges, dual-specificity and multi-specificity targeting strategies are increasingly being explored to simultaneously modulate multiple TME components, thereby enhancing therapeutic selectivity and efficacy. Recent work has demonstrated the feasibility of engineering NPs that bear multiple targeting moieties or hybrid biomimetic coatings. Yao et al. [126] designed a biomimetic platform in which NPs were cloaked with hybrid membranes derived from both CAFs and cancer cells. This design endowed the particles with dual homing capabilities, enabling simultaneous interaction with CAFs and tumor cells. The NPs were loaded with carboplatin and siRNA against p65, allowing them to not only directly kill cancer cells but also suppress CAF-mediated ECM remodeling, ultimately resulting in enhanced intratumoral penetration and improved antitumor efficacy in ovarian cancer models. Similarly, Zhou et al. [127] developed a CAF-targeted nanomedicine for pancreatic cancer therapy in which NPs were functionalized with a peptide ligand (plectin-1 targeted peptide) for tumor cells while being cloaked with CAF membranes to ensure stromal engagement. The system was designed to co-deliver losartan (to degrade the ECM by inhibiting CAF activation) and iron carbonyl (FeCO) for cancer cell killing. This dual-targeting system demonstrated substantial inhibition of tumor growth in vivo, underscoring the advantage of disrupting stromal barriers while simultaneously attacking cancer cells. Despite promising results, the translation of multi-specificity targeting strategies faces several challenges. First, cofunctionalization with multiple ligands may increase synthetic complexity and production costs, which could hinder scalability and regulatory approval [128]. Second, steric hindrance or competition between targeting ligands can reduce binding affinity if not carefully optimized [129]. Third, tumor heterogeneity requires context-specific ligand selection; not all tumors exhibit the same abundance of CAFs, raising questions about the generalizability of dual-targeted designs. Moreover, safety concerns must be considered, as multi-targeted NPs may increase off-target uptake in healthy tissues that express the same receptors [130].

In conclusion, CAF-targeting NPs constitute a multifaceted strategy for modulating the tumor stroma. Through selective cytotoxicity, metabolic interference, immunomodulation, or photoablation, these nanoplatforms offer promising avenues to enhance cancer therapy. Nevertheless, the functional heterogeneity of CAFs highlights the importance of precise, stage-specific targeting to optimize therapeutic outcomes and minimize adverse effects.

## NPs Modulate Tumor ECM

The ECM forms a vital component of the TME, providing not only structural support for epithelial and stromal cells but also playing a crucial role in regulating various cellular functions—including adhesion, differentiation, proliferation, migration, apoptosis, and survival [131]. Additionally, the ECM serves as a reservoir and modulator of bioactive molecules such as growth factors, cytokines, and chemokines, governing their sequestration, activation, and spatial distribution to surrounding cells. While ECM remodeling is tightly regulated under physiological conditions, dysregulation, as frequently observed in cancer, drives disease progression by disrupting cellular signaling and promoting a protumorigenic microenvironment.

Structurally, the ECM consists of a complex network of proteins and polysaccharides, including laminins, collagens, elastin, fibronectin, and proteoglycans—that collectively regulate cell–matrix and cell–cell interactions [132]. It also acts as a reservoir for growth factors, cytokines, and chemokines, modulating their bioavailability and thereby coordinating tissue homeostasis and repair [133]. In cancer, the tumor ECM undergoes substantial remodeling, resulting in increased density, stiffness, and biochemical heterogeneity—factors that substantially influence therapeutic outcomes. A denser ECM creates a physical barrier that hinders immune cell infiltration and restricts the distribution and diffusion of chemotherapeutics and nanomedicines [134]. This limitation is particularly evident in 3D tumor spheroid cultures, where the abundant ECM components form a more physiologically relevant barrier than traditional 2D monolayers, leading to reduced drug penetration and attenuated therapeutic response [135].

Given its dual function as a structural scaffold and a regulator of tumor progression, the ECM represents a crucial target for therapeutic intervention. Dense, dysregulated ECM not only impedes immune cell access but also limits the effective delivery of therapeutic agents into solid tumors. In 3D tumor spheroid models, which better replicate in vivo conditions, drug penetration is significantly reduced compared to 2D cultures—primarily due to the ECM’s high density and architectural complexity [136]. Therefore, targeting the ECM offers a promising strategy to enhance drug delivery and overcome therapy resistance associated with the TME. In particular, NP-based strategies that remodel or exploit ECM components are emerging as effective approaches to facilitate drug transport, modulate tumor–stroma interactions, and improve treatment efficacy [137].

### ECM-mimicking strategies

The ECM presents both structural support and biochemical cues that influence cancer progression. NPs engineered to mimic ECM components aim to exploit these cues for improved targeting and therapy [138]. By resembling natural ECM molecules, ECM-mimicking NPs can enhance cellular recognition, promote specific interactions with tumor cells or stromal components, and facilitate deeper tumor penetration [133]. Biomimetic NPs can also provide a favorable microenvironment for sustained drug release or immune modulation, effectively increasing therapeutic efficacy while minimizing off-target toxicity. Some designs incorporate ECM-derived peptides or surface coatings to mimic ligand–receptor interactions, enhancing adhesion and retention within tumor tissue [139].

One promising strategy involves the development of synthetic ECM-mimicking NPs. Laminin, a key ECM glycoprotein, has been utilized to design biomimetic peptides capable of self-assembling into NPs through hydrophobic interactions. As illustrated in Fig. 4D, Hu et al. [140] introduced a laminin-mimicking peptide that self-assembled into NPs, effectively inhibiting tumor invasion. These laminin mimetics exhibited an intrinsic ability to self-organize into NPs via hydrophobic interactions. Upon binding to laminin receptors and integrins on the surface of cancer cells, the NPs transformed into nanofibers. Functioning as components of an artificial ECM, the engineered laminin-mimicking NPs demonstrated remarkable temporal stability, remaining at tumor sites for up to 3 d. This prolonged retention led to effective suppression of lung metastases across various solid tumor models.

An emerging biomimetic strategy involves engineering cell-adhesive patterns that recapitulate the architecture of the tumor ECM by employing magnetic nanocarriers assembled on agarose hydrogels under controlled magnetic fields. Magnetostatic guidance enabled the formation of both fibrous and mesh-like nanostructures, which effectively directed cell adhesion and migration while promoting osteoclast aggregation. These studies underscore the potential of biomimetic nanomaterials to modulate the ECM, offering a versatile platform to regulate cellular interactions and impede metastatic progression [141]. These ECM-mimicking strategies provide a dual benefit: stabilizing tumor ECM while enhancing NP retention and cellular interactions within the TME.

### Degrading ECM strategies

#### Targeting HA networks

HA is a large, linear glycosaminoglycan composed of repeating disaccharide units of glucuronic acid and N-acetylglucosamine. It is synthesized by hyaluronan synthases (HAS1, HAS2, and HAS3) and primarily degraded by hyaluronidases (HYAL1 and HYAL2) [142]. Under normal physiological conditions, HA levels are tightly controlled through a balance between its production and breakdown. In many cancers, however, HA is often overproduced or accumulates at high concentrations within tumor cells and their surrounding ECM [143]. HA plays a critical role in promoting metastasis and cancer cell proliferation by restricting immune cell infiltration and reducing tumor perfusion while also increasing the likelihood of vascular collapse within the TME. Elevated HA levels exacerbate hypoxia and facilitate immune evasion by impairing vascular function and immune cell access [144].

To counteract these effects, Zhou et al. [145] developed PLGA-PEG NPs loaded with doxorubicin (DOX) and conjugated to recombinant human hyaluronidase PH20. This enzyme degrades HA within the TME. The surface modification led to a 4-fold increase in the accumulation of PLGA-PEG NPs in a 4T1 mouse breast tumor model and promoted their uniform distribution across the tumor. Enzymatic degradation of HA also reduced ECM stiffness, thereby improving immune infiltration and DOX accessibility (Fig. 4F). This approach demonstrates that targeting HA networks can improve NP penetration, reduce ECM barriers, and enhance therapeutic response.

#### Modulation of collagen networks

Collagen is a major structural component of the ECM and plays a critical role in maintaining tissue rigidity, regulating cell adhesion, and influencing tumor progression. Elevated collagen deposition in the TME has been associated with increased resistance to therapies, impaired drug penetration, and enhanced metastatic potential [146]. Therefore, strategies aimed at modulating collagen networks have emerged as a promising approach to overcome ECM-mediated barriers and improve the delivery and efficacy of nanomedicine-based cancer therapies [147].

Collagen is a prominent constituent of the ECM within the TME of malignant tumors. Elevated collagen expression has been linked to resistance to therapeutic interventions and reduced drug absorption [148]. Goodman et al. [149] conjugated collagenase with polystyrene vehicles to degrade collagen in ECM. The influence of NP’s size and collagenase treatment on the penetration of carboxylated polystyrene NPs was evaluated using a multicellular spheroid model. Treatment of spheroids with collagenase markedly enhanced the infiltration of NPs up to 100 nm, while NPs exceeding 100 nm experienced only a slight improvement. To achieve localized ECM degradation, collagenase was immobilized on the surface of NPs. These collagenase-functionalized 100-nm NPs achieved a 4-fold increase in delivery to the spheroid core compared with unmodified NPs. These findings suggest that incorporating ECM-modulating enzymes into NP formulations can substantially enhance their penetration and delivery to solid tumors.

Similar to HA degradation, NPs have been developed in combination with collagen depletion to improve drug delivery. As shown in Fig. 4E, Pan et al. [150] formulated a thermosensitive hydrogel using a PLGA-PEG-PLGA polymer matrix. This hydrogel incorporated the mAb trastuzumab, specifically targeting the HER2 receptor, along with collagenase for peritumoral administration. Application of this gel reduced collagen density and increased apoptotic cell death in the tumor tissue. As a result, this approach showed superior therapeutic outcomes, marked by greater effectiveness and reduced toxicity compared to control groups. Notably, a one-fourth dosage of the gel demonstrated stronger antitumor efficacy than the intravenous administration of the standard clinical trastuzumab formulation. This localized co-delivery system offers a promising strategy for modulating the densely packed ECM and enhancing the efficacy of antibody-based treatments.

Recent advances in understanding the mechanics of native tumor ECM assembly have inspired innovative strategies aimed at intervening during various stages of ECM deposition to correct structural abnormalities. Grossman et al. [151] utilized advanced microscopy techniques to monitor collagen assembly within a 3D matrix. They discovered that antibodies targeting lysyl oxidase-like 2 (LOXL2) could modulate the size and alignment of endogenous collagen fibers while preserving the ECM’s overall composition. These altered collagen morphologies disrupted adhesion and invasive behaviors in human breast cancer cells. Furthermore, the LOXL2-inhibiting antibodies reduced the formation of neoplastic collagen superstructures by 5-fold and decreased tumor growth by approximately 50% in murine breast cancer models. Overall, targeting collagen thus improves NP penetration and antibody therapy while directly modulating ECM-mediated barriers to enhance antitumor efficacy.

### Modulating ECM signaling

Scientific studies have shown that numerous growth factors and signaling molecules released by tumor cells play active roles in ECM deposition, suggesting potential strategies for normalizing the TME by regulating these factors [152]. TGF-β is a prominent example, as it promotes the production of proteases by CAFs, thereby facilitating ECM protein synthesis and remodeling [153]. Building on these insights, Dai et al. [154] developed a multifunctional NPs, PPD/PHDP@siTGF-β, designed with pH-responsive size reduction, charge reversal, and ROS-sensitive degradable properties to deliver siTGF-β for tumor oxidation–chemotherapy combined with TME remodeling. Immunofluorescence analysis revealed the impact of this system on proteins associated with tumor metastasis, angiogenesis, and ECM deposition, including TGF-β, CD31, α-SMA, collagen, and HA. Notably, the reduction of ECM components induced by the nanosystem led to a significant decrease in interstitial fluid pressure (IFP) by nearly 55% and promoted ECM remodeling, which in turn enhanced the accumulation of the NPs within tumor tissues. Overall, this engineered NPs effectively overcame physiological barriers, improved the bioavailability of its cargo with excellent biosafety (maintaining body weight and causing no detectable tissue damage), and enhanced antitumor immune responses through TME modulation. These effects collectively resulted in potent suppression of tumor growth and metastasis, reducing the number of metastatic nodules to approximately ^1^/_6_ of the control.

Matrix metalloproteinases (MMPs), particularly MMP-2, are a group of proteolytic enzymes responsible for degrading various components of the ECM [155]. MMPs are known to contribute to tumor invasion, neoangiogenesis, and metastasis, making them common targets in cancer treatment [156]. The use of MMPs aims to achieve targeted drug delivery and favorable therapeutic outcomes for tumors. Vaghasiya et al. [157] developed mesoporous silica NPs (MSNs) responsive to MMP-2 by loading them with cisplatin (Cis). In this delivery method, collagen was applied to the surface of cisplatin-loaded MSNs (Cis-MSNs) to form a binding layer, resulting in collagen-coated MSNs, referred to as Cis-col-MSNs. Under normal intracellular conditions, the collagen capping prevents Cis molecules from being released from Cis-col-MSN. However, overexpressed MMP-2 in the TME degrades the collagen layer, allowing drug release from MSN pores specifically in the TME. Cellular uptake and cytocompatibility studies using A549 adenocarcinoma lung cancer cell lines showed that the nanocarrier was efficiently internalized within 24 h and exhibited biocompatibility. The therapeutic efficacy of the system was substantially enhanced by ROS generation, cell cycle arrest, and apoptosis. Overall, this NP-based system offers a viable strategy for constructing smart drug delivery platforms that selectively release therapeutic agents at the tumor site.

Because signals from cancer cells can dynamically influence ECM characteristics, targeting intracellular signaling pathways represents a promising approach for ECM modulation [158]. Jiang and colleagues [159] developed a strategy to normalize the TME by simultaneously inhibiting tumor cell signals associated with ECM formation. Specifically, their nanosystem was designed to dual-target key regulators of ECM modulation—the cell surface adhesion receptor integrin and the EGFR. This approach effectively reduced the expression of proteins involved in ECM and TME formation, including α-SMA, TGF-β, collagen, and F-actin, resulting in substantial TME remodeling, as evidenced by approximately a 30% reduction in IFP, and a 40% decrease in solid stress. These changes were accompanied by strong suppression of tumor growth, achieving roughly a 10-fold inhibition rate.

Overall, these strategies underscore the transformative potential of ECM-targeted nanomedicine in cancer therapy. Whether through structural mimicry, enzymatic degradation, or physical remodeling, NP-based interventions provide a multifaceted approach to reprogram the tumor stroma, enhance drug delivery, and overcome therapeutic resistance. Future progress will benefit from deeper insights into ECM dynamics and their interactions with immune and tumor cells, facilitating the development of personalized, ECM-adaptive nanotherapeutics for clinical translation.

## NPs Target Tumor Vasculature

The TME in many solid tumors features a highly heterogeneous tumor vasculature, contributing to irregular blood perfusion within the tumor. This irregularity results in inadequate blood supply to certain tumor regions [160]. Moreover, the absence of functional intratumoral lymphatic vessels further worsens the condition, hindering the effective transport of therapeutic agents to the TME [161]. The blood and lymphatic vascular networks within tumors can obstruct immunosurveillance mechanisms and suppress the antitumor immune response. As a result, innovative therapeutic strategies targeting the restructuring of these stromal elements hold considerable promise for overcoming resistance to immunotherapy [162].

The structural and functional organization of the tumor vasculature plays a critical role in promoting a protumorigenic and immunosuppressive TME [162]. The vascular system acts as a conduit for delivering essential components such as nutrients, oxygen, growth factors, and metabolic by-products, exerting a substantial influence on tumor recurrence, metastasis, and therapeutic resistance. Studies have highlighted the substantial role of tumor vasculature in modulating treatment efficacy by affecting both drug delivery and the availability of key nutrients and oxygen [163].

**Fig. 5.**
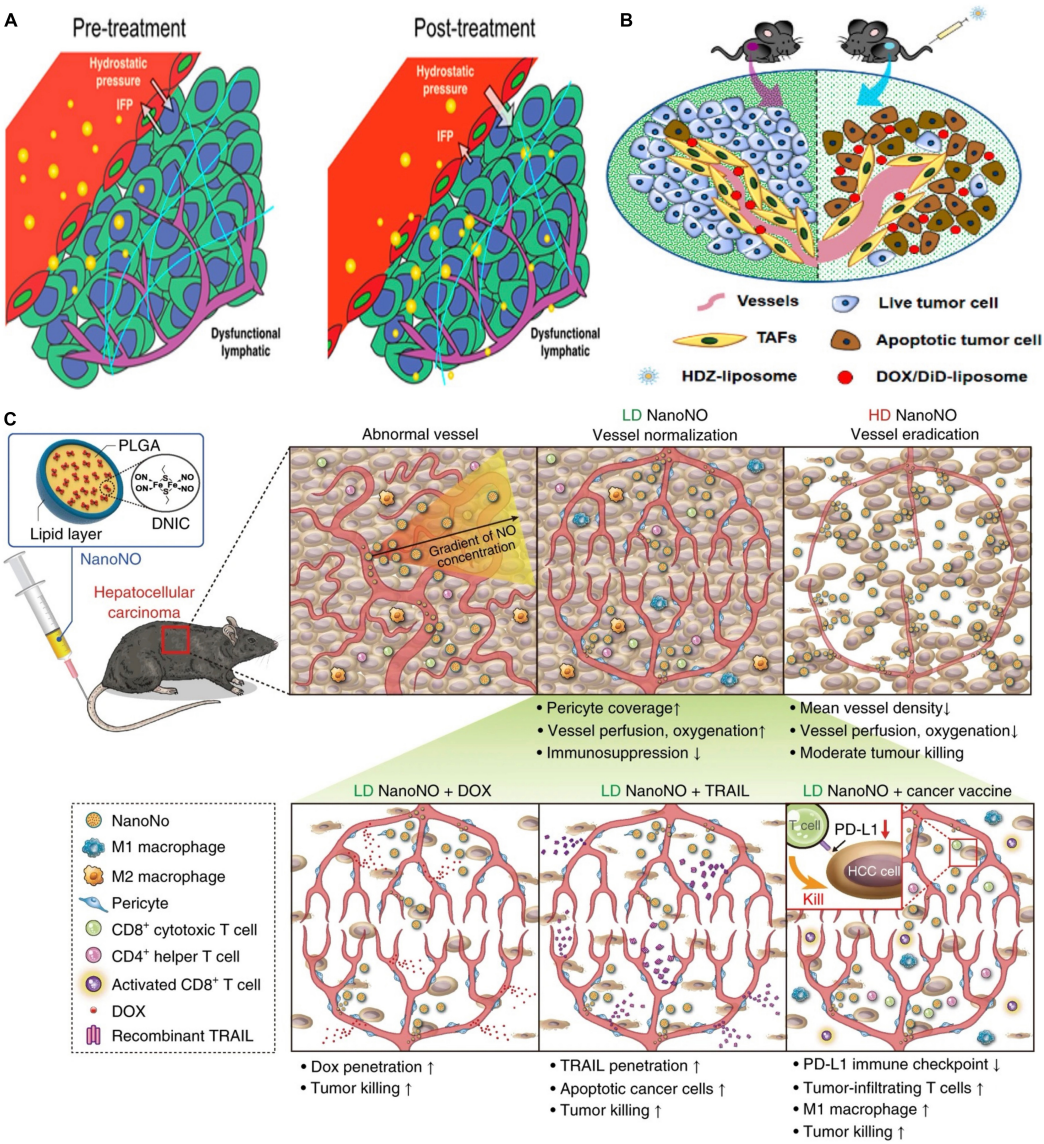
Nanomaterials modulate tumor vasculature and enhance therapeutic efficacy in cancer immunotherapy. (A) Tumor vascular normalization restores pressure gradients and improves perfusion, enabling enhanced delivery of intermediate-sized NPs (20 to 40 nm), and supporting a 2-stage transport strategy for optimal intratumoral distribution. Reproduced with permission from [166]. Copyright 2015, American Chemical Society. (B) The HDZ–liposome system, designed for tumor-selective HDZ delivery, substantially reduced tumor stroma and hypoxia in murine desmoplastic melanoma (BPD6), while reshaping the immunosuppressive microenvironment to enhance immunotherapy potential. Reproduced with permission from [175]. Copyright 2019, American Chemical Society. (C) Development of NanoNO, a nanoscale nitric oxide (NO) delivery system, ensures prolonged half-life and sustained release, enabling efficient NO delivery into hepatocellular carcinoma. Reproduced with permission from [177]. Copyright 2019, Springer Nature.

Normalizing both the morphological and functional features of tumor vasculature is essential for improving tissue perfusion, thereby enhancing intratumoral delivery of NPs [164]. However, this vascular remodeling can also reduce tumor vessel permeability, potentially limiting NP transport. While NPs smaller than 10 nm derive the greatest benefit from vessel normalization therapy in terms of enhanced delivery, their clinical use is constrained by their extremely small size [165]. Interestingly, Jiang et al. [166] demonstrated that NPs of intermediate size (20 to 40 nm) can also exploit tumor vascular remodeling. By applying antiangiogenic therapy targeting VEGF receptor-2 (VEGFR-2) to normalize tumor blood vessels, they substantially enhanced the transvascular delivery of NPs up to 40 nm. These findings suggest that combining antiangiogenic therapy with thoughtful NP design may offer a synergistic, multi-stage approach utilizing distinct size-inclusion strategies for optimizing nanomedicine delivery into solid tumors (Fig. 5A). Additionally, signaling by VEGF and basic fibroblast growth factor (bFGF) in endothelial cells suppresses the expression of adhesion molecules, thereby impairing T cell extravasation into tumors. Strategies to normalize tumor vasculature or inhibit angiogenic signaling have emerged as effective approaches to improve T cell access [167]. Lee et al. [168] developed a multifunctional micellar delivery system based on cationic poly(2-(dimethylamino)ethyl methacrylate)-block-poly(ε-caprolactone) (PDMA-b-PCL) micelles. This platform co-delivered VEGF-targeting siRNA, ultrasmall superparamagnetic iron oxide NPs (USPIOs), and the chemotherapeutic agent SN-38. By combining VEGF silencing with chemotherapy, the system substantially suppressed tumor progression in vivo.

A multifunctional lipid-based NP system developed by Du et al. [169] incorporated low molecular weight heparin (LMWH), gemcitabine, and PTX. LMWH functions by inhibiting the binding of VEGF to its receptors on endothelial cells, thereby blocking the VEGF signaling pathway. Gemcitabine promotes tumor vessel normalization through a metronomic approach, involving the administration of low doses at high frequency. Upon administration, this NP system degrades in the acidic TME, enabling efficient release of gemcitabine and PTX. Enhanced flow perfusion was observed. Fluorescence microscopy of tumor sections from the NP-treated group revealed a transition from irregular tumor vasculature to a more regular pattern during the normalization window. Subsequently, these vessels were degraded, resulting in a nearly 3-fold reduction in blood vessel density.

Beyond VEGF inhibition, vasculature-disrupting agents (VDAs) represent another class of drugs capable of destabilizing existing tumor blood vessels, inducing necrosis in the tumor core due to reduced blood supply [170]. However, tumor cells located at the periphery, where the effects of VDAs are less pronounced, may survive and migrate. This limitation can be addressed through the synergistic use of VDAs with immunotherapy, which can remodel the TME in peripheral tumor regions. Seth et al. [171] designed PLGA NPs loaded with the VDA 5,6-dimethylxanthenone-4-acetic acid (DMXAA) and the TLR7/8 agonist gardiquimod. DMXAA induces endothelial cell apoptosis through TNF-α-mediated pathways. Although both DMXAA and gardiquimod are nontoxic to tumor cells individually, their combined administration produces synergistic tumor-suppressive effects. Gardiquimod activates immune responses via the MyD88 and nuclear factor κB (NF-κB) signaling cascades, while DMXAA facilitates the release of necrotic tumor debris, which serves as a source of TAAs, thereby amplifying the immune response. In vivo studies using a melanoma model demonstrated that this combination strategy resulted in pronounced vascular disruption and a substantial increase in APC infiltration, leading to a significantly improved survival rate of 63.6%, compared to 18.1% with PLGA monotherapy and only 9% with DMXAA alone. These findings underscore the therapeutic potential of combining vascular disruption with immune activation. Notably, previous research has shown that copper promotes angiogenesis by enhancing endothelial cell migration and invasion. The integration of antiangiogenic strategies with immunotherapy in this context yielded synergistic effects, resulting in slower tumor progression and a marked reduction in tumor size (200 mm^3^ compared to 1,500 mm^3^ in the control group) in breast cancer models.

Other studies have focused on improving nanomedicine delivery through antiangiogenic agents. Zhou et al. [172] developed a polymeric NP system encapsulating the EGFR inhibitor erlotinib and DOX. This nanoplatform targets tumor tissue via the enhanced EPR effect, enabling a sustained and controlled release of DOX in combination with erlotinib. The application of antiangiogenic agents substantially enhanced the synergistic efficacy of this combined nanomedicine and chemotherapy approach.

Dysfunctional tumor vasculature impedes the effective delivery and distribution of chemotherapeutic agents, resulting in reduced drug concentrations within the TME and diminished therapeutic efficacy. Normalizing tumor vasculature can regulate vascular permeability, mitigate hypoxia, enhance blood perfusion, lower IFP, and improve drug delivery. Xiao et al. [173] introduced an innovative strategy combining cediranib, a tumor vessel-normalizing agent, with an enzyme-responsive gold NP system (AuNPs-A&C). Their findings showed that pretreatment with cediranib significantly increased tumor vascular permeability and oxygenation while reducing microvessel density by 3-fold, indicating successful vascular normalization and a more favorable TME. Consequently, this combined approach markedly enhanced the tumor-targeting capacity of various NP formulations. Coadministration of cediranib and AuNPs-A&C led to a 1.5-fold increase in tumor permeability, a 1.6-fold improvement in tumor-targeting efficiency, and approximately 70% tumor volume reduction compared to the saline-treated group in 4T1-bearing mouse models.

Although NPs leverage the EPR effect for tumor accumulation, elevated IFP and irregular vasculature—especially in desmoplastic tumors—pose major challenges to deep tissue penetration [174]. To address poor perfusion in desmoplastic tumors, Chen et al. [175] developed liposomal NPs encapsulating hydralazine (HDZ), a well-known antihypertensive agent, to remodel tumor vasculature in advanced melanoma. Intravenous administration of HDZ-liposomes effectively induced vascular dilation, alleviated hypoxia, and increased vascular permeability, thereby reshaping the TME. This vascular modulation substantially improved the antitumor efficacy of liposomal DOX when used as a second-line therapy in mice with tumors exceeding 400 mm^3^ (Fig. 5B).

Moreover, elevated concentrations of nitric oxide (NO) in the perivascular space have been shown to promote the normalization of tumor blood vessels, thereby improving the efficacy of chemotherapy. However, despite its promising anticancer potential, the clinical application of NO remains limited due to its rapid degradation, poor bioavailability, and insufficient tumor-specific delivery [176]. To overcome these challenges, Sung et al. [177] developed a PLGA-based NP system, termed NanoNO, which incorporates a dinitrosyl iron complex as a sustained-release NO donor. In a mouse model of hepatocellular carcinoma, NanoNO facilitated prolonged NO release within tumors, leading to improved vascular normalization and enhanced delivery of chemotherapeutic agents, thereby suppressing both primary tumor growth and metastasis. Further immunological analyses revealed that even at lower doses, NanoNO could modulate the immunosuppressive TME, amplifying the overall anticancer response (Fig. 5C).

Collectively, these findings emphasize the critical role of vascular targeting in enhancing the efficacy of nanomedicine. By modulating the structure and function of tumor blood vessels through normalization, disruption, or remodeling, NP-based strategies can substantially improve drug penetration, immune cell infiltration, and overall therapeutic outcomes. Future studies integrating vascular-targeting NPs with immunotherapy and precision medicine approaches hold promise for overcoming current limitations in the treatment of solid tumors.

## NPs Regulate Tumor Mediators

The TME constitutes a complex and dynamic ecosystem that plays a pivotal role in cancer progression, immune evasion, and therapeutic resistance [6]. This environment is typically categorized into 3 main components: (a) the cellular compartment, comprising immune and stromal cells; (b) soluble mediators, including cytokines, enzymes, and metabolites; and (c) the ECM [8]. In this context, “tumor mediators” refer to the diverse biochemical signals and molecules, both soluble and cell-associated, that actively regulate tumor growth, immune suppression, angiogenesis, metastasis, and other hallmarks of cancer. These mediators include immunosuppressive cytokines or chemokines (e.g., TGF-β and IL-10), metabolic enzymes [e.g., indoleamine 2,3-dioxygenase (IDO)], reactive metabolites [e.g., glutathione (GSH)], and matrix-modifying proteins (e.g., MMPs) [178–180]. Collectively, they create a biochemical milieu that supports tumor survival while limiting immune cell infiltration and therapeutic efficacy [181]. Within this intricate setting, numerous signaling pathways orchestrate tumor cell survival, proliferation, angiogenesis, immune suppression, and metastasis. Several biochemical and physiological characteristics—such as hypoxia, acidosis, elevated levels of immunosuppressive enzymes, and soluble factors like IDO, TGF-β, GSH, and MMPs, collectively foster an immunosuppressive TME that impairs immune cell activity and undermines the effectiveness of anticancer therapies [182]. NPs offer a promising strategy to modulate these tumor mediators, thereby reshaping the TME to enhance immune function and improve therapeutic outcomes.

A key contributor to immune suppression in the TME is IDO, an intracellular, heme-containing enzyme that catalyzes the degradation of l-tryptophan (Trp) into l-kynurenine (Kyn). Many tumors overexpress IDO, resulting in tryptophan depletion that hampers cytotoxic T cell proliferation and promotes immune evasion. This mechanism has spurred the development of NP-based approaches to inhibit IDO activity [183]. Consequently, researchers have sought to synergize the therapeutic benefits of IDO blockade with the advantages offered by NPs to enhance antitumor responses and extend survival [184]. As illustrated in Fig. S1A, Liu et al. [185] engineered IDO-encoded siRNA NPs coated with tyrosinase-related protein 2 (Trp2), expressed in recombinant *Saccharomyces cerevisiae* (YCP). Positively charged and approximately 5 nm in size, YCPs were selectively taken up by immune cells. The YCP-delivered siRNA and Trp2 effectively evaded phagosomal degradation and suppressed IDO expression in DCs, thereby boosting T cell-mediated immune responses against Trp2. This was evidenced by elevated secretion of proinflammatory cytokines, including IFN-γ, TNF-α, and IL-6, along with a reduction in Treg generation. Furthermore, the study demonstrated that YCPs effectively suppressed melanoma tumor growth by reducing immune tolerance and eliciting a Trp2-specific CD8^+^ T cell immune response. In a separate investigation, a novel polymeric IDO inhibitor was developed for cancer immunotherapy. This inhibitor is based on a copolymer of poly(ethylene glycol)-b-poly(l-tyrosine-co-1-methyl-d-tryptophan), designated as PEG-b-P(Tyr-co-1-MT). The block copolymer PEG-b-P(Tyr-co-1-MT) is capable of self-assembling into NPs. These NPs demonstrated the ability to inhibit the metabolic conversion of Trp into Kyn in B16F10 cancer cells. When administered in the form of DOX-loaded NPs to melanoma-bearing mice, the treatment substantially increased the number of mature DCs, CD8^+^ T cells, IFN-γ, and TNF-α while concurrently reducing Treg levels and down-regulating PD-L1 expression. Collectively, these effects contributed to TME modulation, tumor growth suppression, and improved survival outcomes [186].

Another critical component of the TME is oxidative stress, primarily driven by elevated levels of ROS. These increased ROS levels are a hallmark of neoplastic cells and tissues. The accumulation of ROS within the TME induces oxidative stress, triggering an up-regulation of ROS scavengers such as GSH [187]. GSH plays a pivotal role in maintaining the cellular redox balance and provides protection against damage caused by ROS and xenobiotic agents. Interestingly, GSH can exert both protective and pathogenic effects; its elevated concentration in tumor cells compared to normal tissues is associated with enhanced metastatic potential and resistance to chemotherapy [188]. As shown in Fig. S1B, Zhang et al. [189] developed an NP system composed of camptothecin (CPT) prodrugs and PEG, designed to respond to the high GSH levels in tumor cells. The internalization of these NPs by tumor cells occurs in a time-dependent manner, followed by GSH-mediated intracellular disintegration, which releases CPT and enhances its therapeutic efficacy. Moreover, the prodrug-loaded NPs were shown to promote DC maturation and increase CD8^+^ T cell infiltration into tumors, offering a novel strategy to improve CPT-based cancer therapy.

Nanoscale engineering strategies have been employed to develop ROS-responsive NPs aimed at targeting solid tumors and restoring cellular sensitivity to conventional treatment regimens. To further harness oxidative stress, Banstola et al. [190] designed an innovative ROS-responsive nanosystem. They synthesized a dual-targeted delivery platform for temozolomide (TMZ), engineered to respond to both programmed death-ligand 1 (PD-L1)-expressing cancer cells and elevated ROS levels, with the goal of enhancing therapeutic outcomes in the hypoxic TME. Elevated ROS concentrations triggered the release of TMZ from anti-PD-L1-targeted NPs, inducing oxidative damage and mitochondrial-mediated apoptosis. This advanced anti-PD-L1 TMZ-TK NP system exhibited potent antitumor activity, including DNA damage and inhibition of angiogenic markers, offering a versatile and broadly applicable approach to cancer therapy (Fig. S1C).

Hypoxia represents another hallmark of the TME, contributing to tumor progression and metastasis [191]. Although direct oxygen delivery holds conceptual appeal, achieving efficient oxygen loading and delivery via NPs remains a major challenge. As an alternative, nanocarriers can deliver oxygen-generating agents, such as metformin, MnO_2_, or catalase that react with endogenous hydrogen peroxide (H_2_O_2_) to produce oxygen in situ. Among these, MnO_2_-based nanoplatforms have attracted considerable attention [192]. Building on this approach, Wang et al. [193] developed a multifunctional nanoplatform using hollow polydopamine (HPDA) coated with MnO₂, forming HPDA@MnO2@Ce6/DOX@PEG-RGD (HPMRCD) NPs. These particles were functionalized with arginine-glycine-aspartic acid (RGD) peptides for tumor targeting and coloaded with DOX and chlorin e6 (Ce6). Under acidic and oxidative conditions in the TME, the NPs released both therapeutic agents. Concurrently, MnO_2_ reacted with H_2_O_2_ to generate oxygen, thereby enhancing the efficacy of PDT. This platform delivered synergistic benefits by integrating chemotherapy, oxygen generation, and PDT for robust tumor suppression (Fig. S1D).

In general, these studies demonstrate that NP systems can be strategically engineered to modulate key components of the TME, including immunosuppressive enzymes, oxidative stress regulators, and hypoxia-driven pathways. By incorporating targeted delivery, stimulus-responsive release mechanisms, and immunomodulatory functions, these nanoplatforms offer a powerful strategy to reprogram the TME and enhance the effectiveness of cancer therapies.

## Clinical Translation of Nano-Immunotherapies in TME Modulation

In recent years, there has been a notable rise in clinical trials investigating nanotechnology-enabled immunotherapies designed to reprogram the TME and improve therapeutic efficacy [2]. As outlined in Table 1, current candidates in development include liposomes, exosomes, albumin NPs, LNPs, metal-based NPs, and protamine-formulated mRNA platforms. These studies have collectively provided encouraging signals, demonstrating not only the feasibility of integrating nanotechnology with immunotherapy but also the potential to elicit durable immune responses. At the same time, their outcomes highlight both the opportunities and the remaining limitations of this strategy. Several trials underscore how nanocarriers can enhance immunotherapeutic activity by concentrating their effects within the TME. For example, Sahin et al. [194] reported that a liposomal RNA vaccine encoding melanoma antigens (BNT111), administered in combination with PD-1 blockade, generated strong antigen-specific CD4^+^ and CD8^+^ T cell responses with durable activity in patients with checkpoint inhibitor-experienced melanoma. Similarly, DC-derived exosomes loaded with MHC-I antigens boosted immune activation in NSCLC, translating into prolonged progression-free survival [195]. Albumin-based formulations such as nab-PTX illustrate an additional advantage: Beyond chemotherapy delivery, they can modulate the TME by depleting immunosuppressive MDSCs and regulatory Tregs, thereby promoting DC function and antigen presentation [196]. In another example, the KEYNOTE-942 trial demonstrated that an individualized mRNA vaccine (mRNA-4157) combined with pembrolizumab significantly improved recurrence-free survival compared with pembrolizumab alone in patients with resected melanoma, reinforcing the translational promise of NP-enabled vaccines [197]. Collectively, these features—precise delivery, protection of therapeutic cargo, and the flexibility to deliver multiple immunomodulating factors—highlight why nanomedicine holds strong potential for overcoming TME-driven immunosuppression.

**Table 1.** Recent NP-based immunotherapies in clinical trials. The following table summarizes NP-based immunotherapies currently under clinical investigation, highlighting their design, mechanisms of action, and targeted cancer types.

NCT number	Nanocarrier	Therapeutics	Phase	Cancer type	TME responses	Clinical benefits	Ref.
NCT02410733	Liposome	BNT111 (mRNA for 4 melanoma antigens) ± anti-PD-1	I	Melanoma	Robust induction of tumor-specific CD4^+^ and CD8^+^ T cell immunity; conversion of “cold” tumors into highly inflamed, immune-infiltrated microenvironments	Durable objective responses and strong T cell responses in ICI-experienced melanoma patients	[194]
NCT01159288	DC-derived exosomes	IFN-γ-matured exosome loaded MHC-II antigen	II	NSCLC	Enhanced innate immunity via activation of NK cells (NKp30-dependent) within the TME	Immune-activating exosomes correlated with prolonged PFS; evidence of NK cell-mediated antitumor activity	[195]
NCT03589339	Hafnium oxide NP	Intratumoral NBTXR3 + radiotherapy + anti-PD-1	I	HNSCC, NSCLC, melanoma	NBTXR3 amplifies radiotherapy-induced immunogenic cell death, increasing local antigen release and promoting an abscopal immune effect	Hypothesized synergy: improved local tumor kills and systemic responses when combined with PD-1 blockade	[199]
NCT02551409	Albumin NP (nab)	Carboplatin + nab-PTX + pembrolizumab	I/II	Advanced NSCLC	Chemotherapy (nab-PTX + carboplatin) can reduce immunosuppressive MDSCs/Tregs and enhance DC antigen presentation	Overall response ~35%; median OS ~15.4 mo. Regimen was safe and showed durable survival outcomes comparable to larger trials	[196]
NCT02819518	Albumin NP (nab)	Pembrolizumab + chemotherapy (taxanes)	III	TNBC	PD-1 blockade combined with NP-based taxanes enhances TILs and antitumor immunity, particularly in PD-L1-positive tumors	Significant improvements in PFS and OS versus chemo alone in PD-L1^+^ TNBC	[225]
NCT01915524	Protamine-formulated mRNA	CV9202 (mRNAs encoding 6 lung cancer antigens) + radiation therapy	Ib	NSCLC	Induction of vaccine-specific T cells against multiple tumor antigens and “antigen spreading” to other tumor epitopes	Demonstrated feasibility of combining mRNA vaccine with radiotherapy; showed immune responses to encoded antigens	[226]
NCT05264974	LNP	Personalized tumor-derived mRNA (neoantigen vaccine)	I	Melanoma	Reprograms TME (bridging innate and adaptive immunity)	Aims to restore anti-PD-1 response (improving relapse-free survival)	[200]
NCT03897881	LNP	Personalized neoantigen mRNA (mRNA-4157) + pembrolizumab	IIb	Melanoma	Induces neoantigen-specific T cells and increases TILs	Improved recurrence-free survival vs. pembrolizumab (HR 0.56)	[197]
NCT03739931	LNP	mRNA-2752 (OX40L, IL-23, IL-36γ) ± durvalumab	I	TNBC, HNSCC, lymphoma, urothelial	Local IL-23/IL-36γ and OX40L release; activates effector T cells	Increased CD8^+^ T cells and IL-22/IL-6 in TME (biomarkers of response)	[227]
NCT03323398	LNP	mRNA-2416 (OX40L) ± durvalumab	I/II	Ovarian cancer	Up-regulates OX40L costimulation; increases T cell infiltration	Boost T cell-mediated tumor killing	[228]
NCT01095848	Lipid depot (liposome-like)	Multi-peptide vaccine (DPX-0907) + GM-CSF	I	Breast, ovarian, prostate cancer	Elicits strong CD8^+^ T cell and memory responses to tumor antigens	61% of patients had antigen-specific T cell responses	[229]
NCT03606967	Albumin NP (nab)	Nab-PTX + durvalumab + tremelimumab + neoantigen peptide vaccine + poly-ICLC	II	TNBC	Chemotherapy/ICB + vaccine to deplete suppressors and prime effector T cells	Evaluates added benefit of neoantigen vaccine on ORR/PFS	[230]
NCT03289962	LNP	Personalized mRNA neoantigen vaccine + atezolizumab	I	Melanoma, NSCLC, CRC	Induces polyclonal neoantigen-specific T cells; increases CD8^+^ TILs	Induced neoantigen-specific T cell responses in patients	[201]
NCT03376639	LNP	mRNA-2417 (IL-12) ± durvalumab	I	HNSCC	TH1 polarization of TME (increasing IFN-γ, CD8^+^ T cells)	Elicits potent local and systemic antitumor immunity	[231]

HNSCC, head and neck squamous cell carcinoma; HR, hazard ratio; ORR, objective response rate; PFS, progression-free survival; OS, overall survival

Nevertheless, these advances have not consistently translated into transformative clinical benefit. One major barrier is the inherently immunosuppressive TME, characterized by stromal barriers, inhibitory cytokines, and T cell exhaustion, which can limit the effectiveness of even well-designed NP-based delivery systems [198]. Second, limited efficacy reflects suboptimal antigen selection or insufficient immune priming. For instance, early trials of hafnium oxide NPs (NBTXR3) in combination with radiotherapy and PD-1 blockade in head and neck squamous cell carcinoma showed signs of immune activation and abscopal effects but only modest clinical efficacy, requiring further validation in larger studies [199]. Personalized mRNA vaccines also demonstrated feasibility and immunogenicity, but the strength and durability of T cell responses varied considerably between patients [200,201]. Third, manufacturing and safety considerations pose further obstacles. Novel NPs must undergo rigorous biocompatibility testing before human use, leading many clinical studies to focus on established platforms such as liposomes, LNPs, and PEG/PLGA-based polymers [202]. More complex or inorganic systems, including gold or carbon-based nanomaterials, carry additional regulatory scrutiny and are difficult to scale reproducibly [203]. Industrial manufacturing introduces further concerns, as even subtle variations in particle size or drug loading can alter pharmacological properties, emphasizing the need for standardized, cost-effective production pipelines and robust safety protocols [13]. Finally, immune-related toxicities remain a pressing concern. While nanocarriers can confine the activity of potent immune agonists to the tumor site and reduce systemic exposure, risks such as cytokine storm, autoimmunity, or severe adverse events highlight the need for cautious dose optimization and careful selection of combination regimens [204]. These safety considerations, together with the biological and manufacturing hurdles, underscore why translating nano-immunotherapies into routine clinical use remains a complex endeavor [205].

## Conclusion and Future Perspective

Over the past decade, mounting evidence has underscored the TME as a critical driver of cancer progression, immune evasion, and therapeutic resistance, establishing it as a key target in cancer immunotherapy. This review has highlighted the potential of engineered nanomaterials to modulate the TME and address these challenges. An overview comparing the components of the TME targeted by different nanotechnological strategies, along with their mechanisms, advantages, disadvantages, and development stages, is provided in Table S2. Specifically, nanocarriers offer distinct advantages, including tunable surface functionalities, protection of sensitive therapeutic agents from enzymatic degradation, and precise delivery of immunomodulators to specific cellular targets within tumors. Engineered NPs have shown promise in disrupting the ECM and abnormal tumor vasculature, thereby improving drug penetration and retention. Additionally, certain nanoplatforms can reshape the immune microenvironment by reprogramming TAMs, Tregs, DCs, and other immunosuppressive populations to restore effective antitumor immunity. Despite these advances, concerns about immune-related adverse effects remain, necessitating comprehensive preclinical evaluation to ensure the safety and reliability of these emerging therapies.

Looking ahead, several challenges must be addressed to facilitate the successful clinical translation of nanoimmunotherapeutics. One substantial hurdle lies in the complexity of NP design—multifunctional platforms often require elaborate formulations incorporating stabilizers, targeting ligands, and stimuli-responsive elements, which can complicate scalability, increase manufacturing costs, and pose unforeseen toxicological risks [206].

While nanotechnology has yielded several encouraging advances, it is equally important to recognize the complexity and limitations that have hindered broader clinical translation. The design of NPs remains inherently complex, as small changes in size, surface charge, or ligand density can dramatically alter biodistribution, immune recognition, and therapeutic efficacy. Scale-up and batch-to-batch reproducibility are additional barriers that complicate manufacturing and regulatory approval [207]. Safety concerns also remain prominent. Beyond short-term toxicology, long-term biodistribution, persistence in the reticuloendothelial system, and immunogenicity are not yet fully understood. For instance, cationic lipid-based carriers, though potent for nucleic acid delivery, have frequently caused dose-limiting toxicities, while several inorganic nanomaterials, such as gold NPs and quantum dots, raise questions regarding biodegradability and chronic accumulation [208]. Importantly, not all nanomedicine candidates that entered clinical trials have succeeded. Clinical translation has also faced setbacks, with several high-profile failures highlighting the gap between preclinical promise and patient outcomes. For instance, many siRNA- or miRNA-loaded nanocarriers that showed strong efficacy in murine tumor models demonstrated limited therapeutic benefit in human trials, largely due to differences in TME, immune system complexity, and delivery barriers. Notable examples include CALAA-01 (a cyclodextrin-based siRNA delivery system) and BIND-014 (a targeted polymeric NP for docetaxel delivery), both of which showed strong preclinical efficacy but were discontinued due to limited therapeutic benefit and safety/scale-up challenges in human trials [209,210]. These failure cases emphasize the translational gap between animal models and the complex heterogeneity of human tumors, highlighting the necessity for more predictive preclinical models and rigorous long-term safety evaluation. Similarly, some polymeric NPs advanced into early-phase trials but were discontinued due to low efficacy or unacceptable toxicity profiles [211,212]. These cases underscore the need for more predictive preclinical models, improved safety evaluation, and strategies to overcome tumor heterogeneity.

The next generation of NPs should be engineered not only to deliver immunomodulatory agents with spatial and temporal precision but also to dynamically adapt to evolving TMEs. These adaptive platforms may include stimuli-responsive carriers capable of releasing cargo in response to hypoxia, acidity, ROS, or specific enzymatic cues within tumors. In parallel, there must be rigorous evaluation of long-term safety, off-target effects, and immunogenicity to ensure clinical applicability. Additionally, artificial intelligence (AI) and machine learning hold transformative potential to accelerate innovation in nanoimmunotherapy. AI-driven modeling can assist in predicting NP interactions with biological systems, optimizing formulation parameters, and identifying novel combinations of materials for enhanced performance. Furthermore, AI algorithms can analyze large datasets from patient-derived tumors to identify biomarkers predictive of response to nanoimmunotherapy, enabling personalized design of nanoplatforms tailored to individual tumor immune landscapes. Incorporating AI into the design and preclinical validation pipeline could reduce trial-and-error experimentation, expedite discovery cycles, and improve translational outcomes. Consequently, future research should aim to balance therapeutic efficacy with formulation simplicity, manufacturability, and safety. By learning from both the successes and failures of nanotechnology, the field can move toward more realistic, clinically viable strategies. Progress in materials science, bioengineering, and immunology is expected to drive the development of smarter, more efficient drug delivery systems capable of selectively remodeling the TME while minimizing systemic exposure. As theoretical knowledge deepens and engineering technologies evolve, the realization of clinically viable nanomedicine platforms that normalize the TME and synergize with immunotherapy is becoming increasingly feasible. As we are moving into the era of precision medicine, these intelligent and targeted systems hold substantial promise for reshaping the future of precision oncology and advancing the next generation of cancer immunotherapy by overcoming current therapeutic barriers and offering more personalized, effective, and safer treatment options for cancer patients.

## Data Availability

Data will be made available on request.

## Supplementary Materials

Supplementary 1 Graphical Abstract Fig. S1 Tables S1 and S2 References [213–224]
