# Supplementary material for: Engineering Nanoparticles to Modulate Extracellular Matrix and Immune Components of the Tumor Microenvironment in Cancer Immunotherapy
Source: Biomater Res. 2025 Dec 9;29:0289. doi: 10.34133/bmr.0289 (PMC12688472; doi:10.34133/bmr.0289)
Supplement: Supplementary 1 — Graphical Abstract Fig. S1 Tables S1 and S2 References [213–224] [file bmr.0289.f1.zip › Supplemental Materials_Biomaterials research_revised.docx]

***Supplemental Materials***

**Engineering Nanoparticles to Modulate Extracellular Matrix and Immune Components of the Tumor Microenvironment in Cancer Immunotherapy**

Bao-Toan Dang^a,b^, Khang-Yen Pham^c^, Ai Han Nguyen^b^, Jong Jun Park^d^, Taeg Kyu Kwon^e^, Jong-Sun Kang^f^, Jee-Heon Jeong^a^, Simmyung Yook^c,d*^

^a^ Department of Precision Medicine, School of Medicine, Sungkyunkwan University, Suwon 16419, Republic of Korea

^b^ Department of Biomedical Engineering, University of Connecticut, Storrs, CT 06269, USA

^c^ School of Pharmacy, Sungkyunkwan University, Suwon 16419, Republic of Korea

^d^ Department of Biopharmaceutical Convergence, Sungkyunkwan University, Suwon 16419,

Republic of Korea

^e^ Department of Immunology, School of Medicine, Keimyung University, Daegu 42601, Republic of Korea

^f^ Department of Molecular Cell Biology, School of Medicine, Sungkyunkwan University, Suwon 16419, Republic of Korea

**Corresponding author**

*******Simmyung Yook, Ph.D.**

School of Pharmacy and Department of Biopharmaceutical Convergence, Sungkyunkwan University, Suwon 16419, Republic of Korea

E-mail address: [ysimmyung@skku.edu](mailto:ysimmyung@skku.edu)

**Graphical abstract**

**
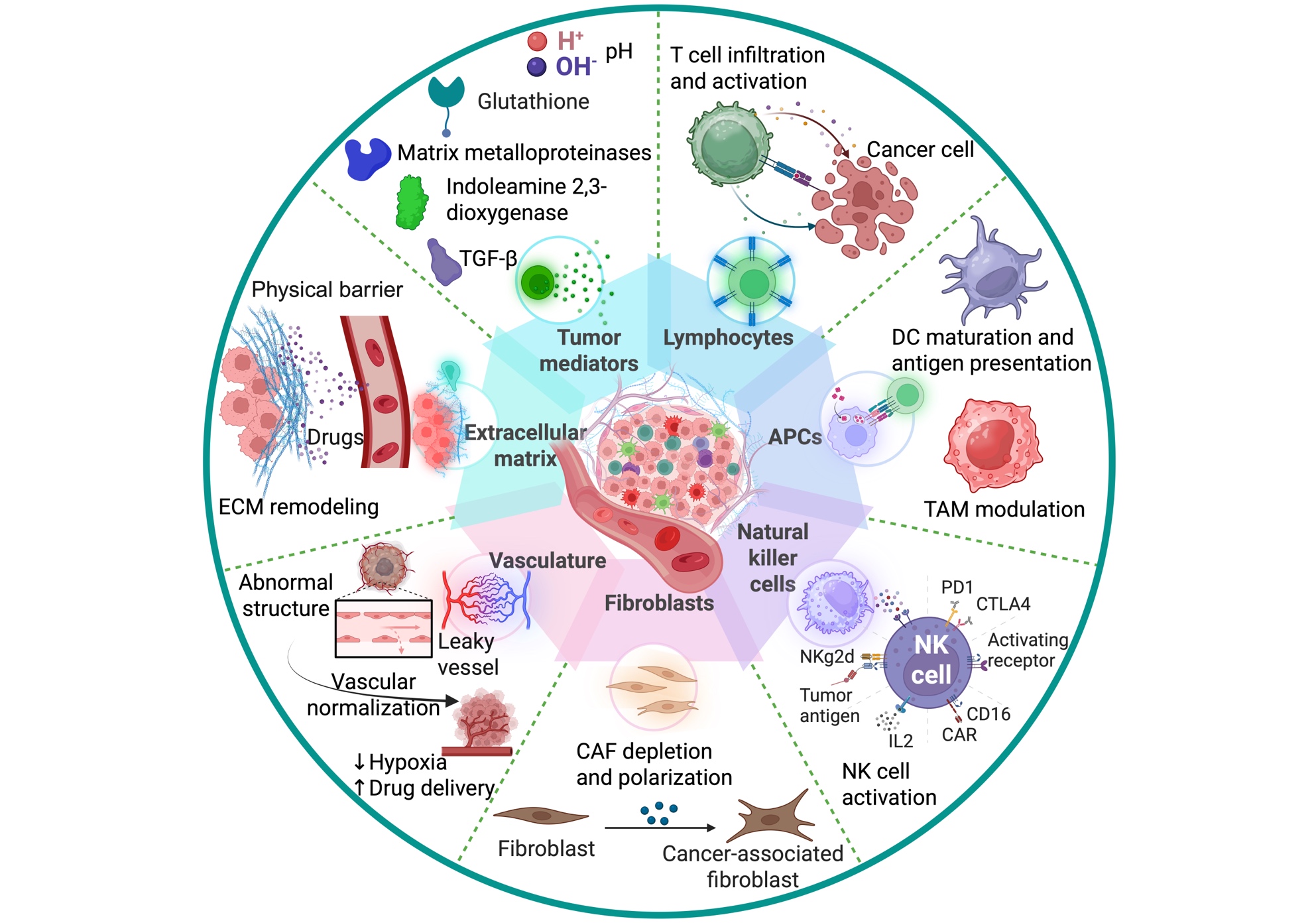
**

**Table S1.** Barriers in the TME that impede cancer immunotherapy.

| **TME barrier** | **Key cells** | **Molecular mediators** | **Mechanisms** | **Impact on immune responses** | **Ref.** |
| --- | --- | --- | --- | --- | --- |
| Fibroblast recruitment and activation | CAFs | TGF-β, FGF, EGF, IL-6, IL-8, IL-17A | - Fibroblasts are activated into CAFs  - CAFs remodel ECM and secrete immunosuppressive cytokines  - Promote tumor growth and immune evasion | - Physical exclusion of T cells  - CAF-secreted factors inhibit DC and T cell function  - Reduced response to ICIs | [220, 221] |
| Immune evasion | M2 TAMs, myeloid cells, Tregs | IL-10, TGF-β, M-CSF, IL-35 | - Polarization toward M2 phenotype supports immunosuppression  - Tregs inhibit CD8⁺ T cells and DCs  - Cytokine secretion dampens immune activation | - Suppression of effector T cell activity  - Reduced antigen presentation  - Resistance to T cell-mediated killing | [222, 223] |
| Suppressive MDSCs | MDSCs | ARG1, iNOS, ROS, IL-10 | - Depletion of L-arginine  - Suppress antigen presentation  - Inhibit cytotoxic T cell responses | - Impairment of antitumor immunity  - Poor outcomes with T cell–based immunotherapies | [224, 225] |
| ECM remodeling | CAFs | Matrix proteins (collagen, fibronectin), TGF-β | - Dense ECM hinders immune cell infiltration  - ECM components disrupt T cell – cancer cell interaction  - Physical and biochemical barriers | - Impaired T cell trafficking and access to tumor cells  - Reduced effectiveness of adoptive cell therapies | [226, 227] |
| Abnormal tumor vasculature | Endothelial cells, pericytes | VEGF, angiopoietins | - Excess VEGF leads to leaky, disorganized vessels  - Abnormal vessels cause poor perfusion and hypoxia  - Barrier to immune cell extravasation | - Inefficient immune cell infiltration  - Decreased delivery of therapeutic antibodies and drugs  - Resistance to immunotherapy | [228, 229] |
| Hypoxia | Cancer cells, stromal cells | HIF-1α, VEGF | - Hypoxia stabilizes HIF-1α  - Increases VEGF expression and ICIs (PD-L1)  - Promotes glycolysis and acidosis | - Immunosuppressive environment  - Upregulation of PD-L1 reduces T cell function  - Promotes exhaustion of immune cells | [230, 231] |
| Immuno-suppressive ligand expression | Cancer cells, APCs | PD-L1, CTLA-4 ligands | - PD-L1 binds to PD-1 on T cells, inhibiting their activity  - CTLA-4 outcompetes CD28 for costimulatory signals | - T cell exhaustion and apoptosis  - Resistance to immune checkpoint blockade | [232, 233] |

**Table S2.** Overview comparing the components of the TME targeted by different nanoparticles, their main mechanisms, advantages and disadvantages, and current stages of development.

| **TME component** | **Nanocarrier** | **Primary mechanisms** | **Advantages** | **Disadvantages** | **Development stage** |
| --- | --- | --- | --- | --- | --- |
| Cytotoxic & effector T cells | Liposomes, PLGA/PEG micelles, polymeric NPs, nano-immuno-conjugates, hydrogel depots | Localized delivery of ICIs, co-delivery of chemo + immune modulators; sustained release depots to reduce systemic exposure; CAR gene delivery *in vivo* | -Enhances T cell activation, lowers systemic dose, can cross barriers.  -Focused ICI reduces systemic immune-related adverse events; enables combination with local modalities | -Complex formulation; off-target immune activation; manufacturability for combinatorial cargos  -Manufacturing complexity; potential for local immune pathology; dose-finding for sustained depots | Preclinical to some early clinical evaluation for liposomal/ICI combos. |
| Dendritic cells (DCs) | Ultra-pH-sensitive polymeric NPs, dendrimers, cell-membrane coated NPs, magnetic NPs | Targeted antigen/adjuvant delivery; STING/TLR activation; enhanced cross-presentation and lymph node trafficking | Strong antigen-specific T cell priming; potential for vaccine-like responses | Need precise targeting; risk of systemic cytokine release; stability of antigen presentation | Mostly preclinical; some vaccine NPs in early clinical stages |
| Natural killer (NK) cells | Supermagnetic NPs, nanoemulsion, exosome, polymeric NPs | Increase NK ligands on tumor cells (MHT), deliver cytokine/siRNA to relieve suppression, exosome miRNA delivery | Enhances innate cytotoxicity; can synergize with other immunotherapies; non-MHC dependent killing | Targeting specificity, potential toxicity from magnetic/metal NPs, gene transfer efficiency | Preclinical; translational interest for exosome and gene-delivery systems |
| Tumor-associated macrophages (TAMs) | PLGA/PEI micelles, iron oxide NPs, nanomicelles | Deliver small molecules or RNAs to repolarize M2→M1 or deplete M2 TAMs (CSF-1R, PI3K-γ inhibition) | Reverses immunosuppression; facilitates T cell infiltration; image-guided theranostics possible | Off-target phagocyte uptake; long-term effects on host macrophage populations; clearance issues | Preclinical to some clinically used iron oxide NPs (ferumoxytol) show immunomodulatory effects |
| Suppressive soluble mediators/ Myeloid-derived suppressor cells (MDSCs) | Liposomal formulations, composite lipid-silica NPs, cytokine-modulating NPs | Deplete/suppress MDSCs or reprogram them into mature APCs; neutralize suppressive factors | Lowers immune suppression, enhances antigen presentation | Complex immune balance; potential systemic cytokine effects; formulation complexity | Preclinical; early translational interest |
| Cancer-associated fibroblasts (CAFs) | Ferritin NPs; CAP peptide NPs; targeted liposomes | -CAF ablation (photoablation/cytotoxic payload), FAP-mediated drug release, metabolic inhibition of CAF support | Reduces stromal barrier; improves penetration and T cell access | CAF heterogeneity (some CAF subsets tumor-suppressive); off-target fibroblast damage; safety concerns (e.g., sibrotuzumab) | Preclinical to some clinical antibody trials highlighted safety risks |
| Extracellular matrix (ECM) | PLGA–PEG NPs, hyaluronidase NPs, collagenase NPs; collagenase depots; MMP-responsive MSNs | -HA degradation to reduce stiffness/IFP and enhance NP infiltration  -Collagen degradation or crosslinking inhibition to improve penetration  -MMP-triggered release and TGF-β inhibition to normalize ECM | -Dramatically improves intratumoral distribution and drug access  -Improves penetration of NPs up to certain sizes; enhances antibody and drug efficacy  -Tumor-selective release, reduces IFP and solid stress, improves NP accumulation | -Enzyme stability; risk of invasion/metastasis; immunogenicity  -Tissue damage; metastasis risk; local vs systemic control  -Heterogeneous protease expression; off-target activation | Preclinical; concept carried into clinical formulations in other contexts (e.g., rHuPH20 in combination therapies) |
| Tumor vasculature | Micelles, anti-angiogenic codelivery NPs, oxygen-generating NPs, PLGA NPs | Vessel normalization (improves perfusion) or vascular disruption (VDAs) + immune activation; oxygen delivery to relieve hypoxia | Improves drug and immune cell delivery (normalization); VDAs can expose TAAs and synergize with immunostimulants | Normalization window timing; smaller NPs benefit more; VDAs can spare peripheral tumor cells; vascular toxicity risks | Preclinical to early clinical exploration for some NP-based anti-angiogenic approaches |


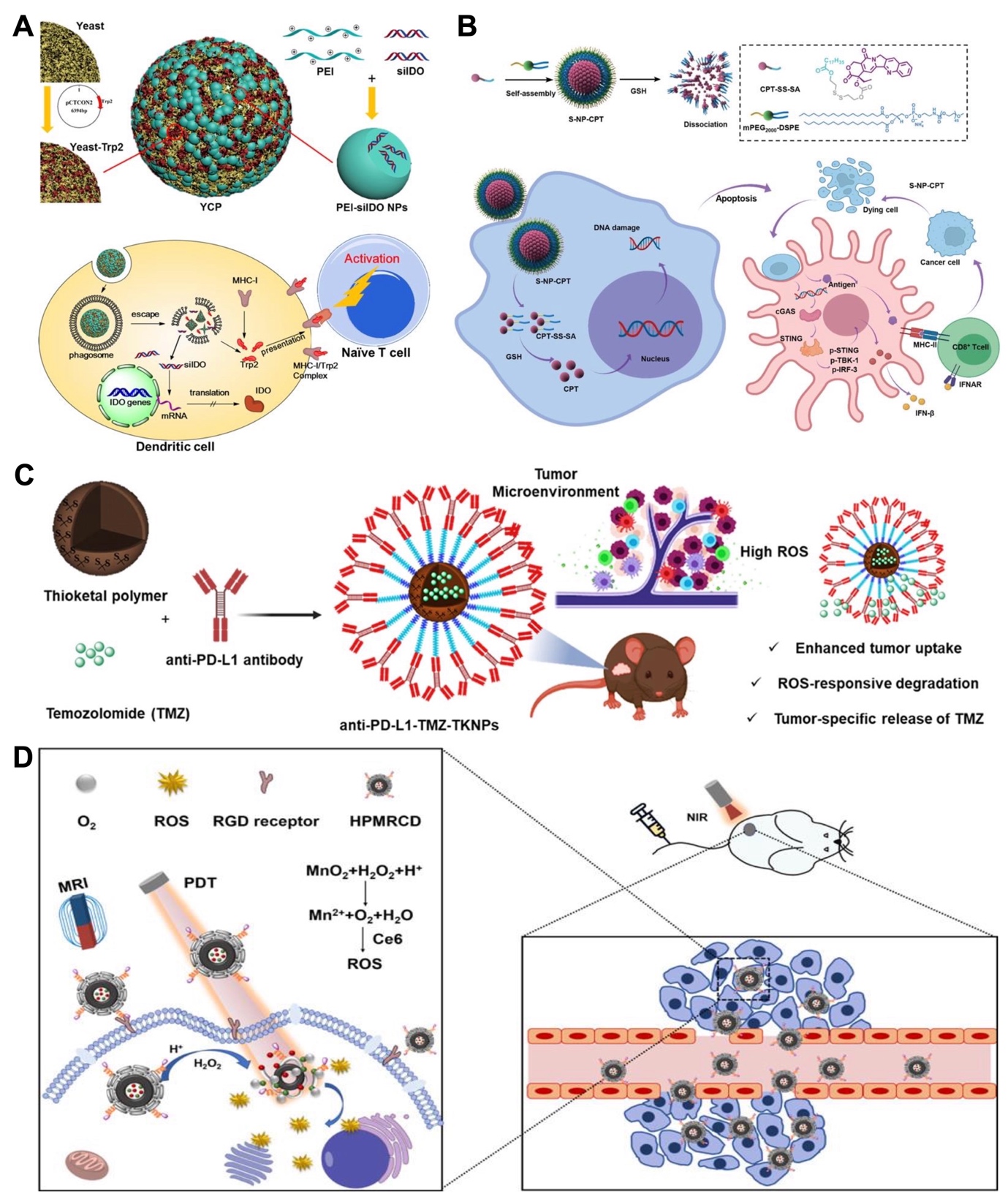


**Figure S1.** NPs modulate TME by targeting pivotal tumor-associated mediators and amplifying immune responses in cancer immunotherapy. **A.** IDO siRNA-coated, tyrosinase-related protein 2-displaying recombinant *Saccharomyces cerevisiae* (YCP) effectively delivers siRNA to DCs, inhibits IDO expression in the TME, enhances T cell-mediated immune responses, increases proinflammatory cytokine secretion, and reduces Treg levels. Reproduced with permission from [234]. Copyright 2018, Elsevier. **B.** A GSH-responsive prodrug system releasing camptothecin, assembled with PEGylated lipids into NPs, provides prolonged circulation, enhanced tumor targeting, and improved therapeutic efficacy. Reproduced with permission from [235]. Copyright 2022, John Wiley and Sons. **C.** A dual-targeted nanosystem encapsulating temozolomide, responsive to both PD-L1 on cancer cells and reactive oxygen species (ROS) within the TME, delivers enhanced anticancer efficacy under hypoxic tumor conditions. Reproduced with permission from [236]. Copyright 2021, American Chemical Society. **D.** Hollow polydopamine NPs coated with MnO₂, functionalized with PEG-RGD, and coloaded with Ce6 and DOX accumulate in tumors and rapidly disassemble in the acidic, H_2_O_2_-rich hypoxic TME. Reproduced with permission from [237]. Copyright 2021, American Chemical Society.
