# Supplementary figures and images for: Engineering Nanoparticles to Modulate Extracellular Matrix and Immune Components of the Tumor Microenvironment in Cancer Immunotherapy
Source: Biomater Res. 2025 Dec 9;29:0289. doi: 10.34133/bmr.0289 (PMC12688472; doi:10.34133/bmr.0289)

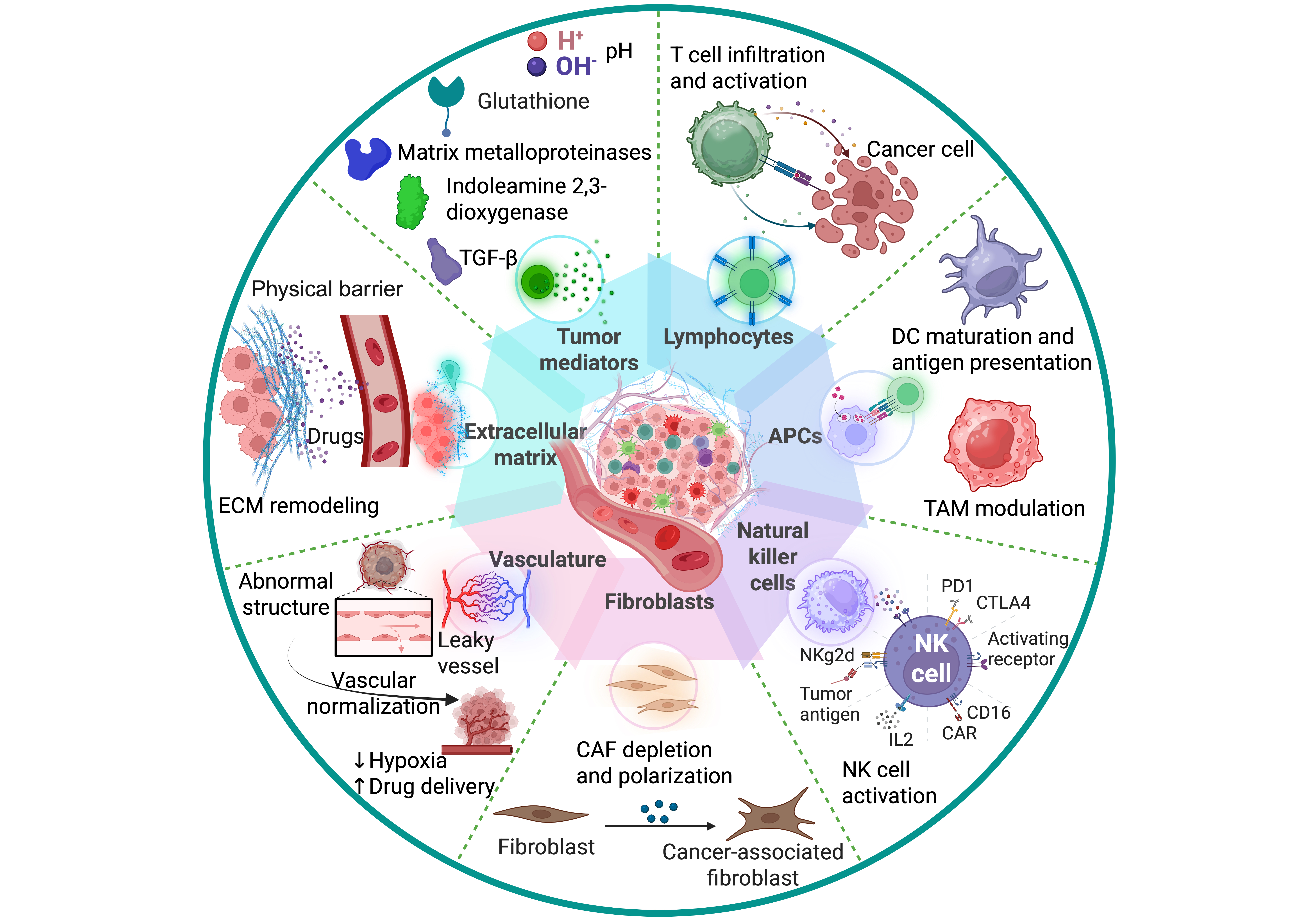

Supplement: Supplementary 1 — Graphical Abstract Fig. S1 Tables S1 and S2 References [213–224] [file bmr.0289.f1.zip › Graphical abstract.jpeg]
